# The costs of being consequentialist: Social inference from instrumental harm and impartial beneficence

**DOI:** 10.1016/j.jesp.2018.07.004

**Published:** 2018-11

**Authors:** Jim A.C. Everett, Nadira S. Faber, Julian Savulescu, Molly J. Crockett

**Affiliations:** a Uehiro Centre for Practical Ethics, University of Oxford, UK; b Department of Experimental Psychology, University of Oxford, UK; c Department of Psychology, Yale University, United States of America

**Keywords:** Morality, Deontology, Consequentialism, Utilitarianism, Impartiality, Person perception, Partner choice, Trust, Prosociality, Helping

## Abstract

Previous work has demonstrated that people are more likely to trust “deontological” agents who reject harming one person to save many others than “consequentialist” agents who endorse such instrumental harms, which could explain the higher prevalence of non-consequentialist moral intuitions. Yet consequentialism involves endorsing not just instrumental harm, but also impartial beneficence, treating the well-being of every individual as equally important. In four studies (total *N* = 2086), we investigated preferences for consequentialist vs. non-consequentialist social partners endorsing instrumental harm or impartial beneficence and examined how such preferences varied across different types of social relationships. Our results demonstrate robust preferences for non-consequentialist over consequentialist agents in the domain of instrumental harm, and weaker – but still evident – preferences in the domain of impartial beneficence. In the domain of instrumental harm, non-consequentialist agents were consistently viewed as more moral and trustworthy, preferred for a range of social roles, and entrusted with more money in economic exchanges. In the domain of impartial beneficence, preferences for non-consequentialist agents were observed for close interpersonal relationships requiring direct interaction (friend, spouse) but not for more distant roles with little-to-no personal interaction (political leader). Collectively our findings demonstrate that preferences for non-consequentialist agents are sensitive to the different dimensions of consequentialist thinking and the relational context.

## Highlights

• We examine perceptions of agents who harm (“instrumental harm”) or help (“impartial beneficence”) for the greater good
• Non-consequentialist agents who refuse to harm for the greater good are consistently preferred across a range of measures
• Consequentialist agents who help for the greater good are perceived as worse friends and spouses, but not political leaders

## 1 Introduction

What unites psychologists, philosophers, and fiction writers? One thing stands out: a fascination with how people do, or should, respond when faced with a decision to sacrifice one innocent person to save a greater number of people. What should one do, for example, if the only way to prevent a major terrorist attack is to torture the child of the suspected terrorist until she releases the information of where her father is? In the academic literature, when someone endorses this harm in such “sacrificial dilemmas” they are typically said to be making a “consequentialist” (or “utilitarian”) judgment in line with consequentialist ethical theories (Bentham, 1789/1983; Mill, 1863). These theories posit that consequences are the *only* thing that matters when making a moral decision - an action is good if it produces good consequences, and bad if it produces bad consequences. In contrast, when someone rejects inflicting harm on an innocent they are said to be making a “non-consequentialist”, or “deontological” judgment in line with deontological ethical theories (e.g. Fried, 1978; Kant, 1797/2002; Rawls, 1971; Scanlon, 1998; W.D. Ross, 1930) positing that even if sacrificing someone to save the lives of five others is an action that maximises overall welfare (“the Good”), this does mean it is morally correct (“the Right”).

Such dilemmas capture our imagination not just because they force an internal moral conflict, but because we recognize the reputational consequences that these impossible decisions might have for those who make them. Recent research has shown that agents who make consequentialist judgments in sacrificial dilemmas are seen as less moral, trustworthy and warm, chosen less frequently as social partners, and trusted less in economic exchanges (e.g. Bostyn & Roets, 2017; Sacco, Brown, Lustgraaf, & Hugenberg, 2017; Everett, Pizarro, & Crockett, 2016; Rom, Weiss, & Conway, 2017; Uhlmann, Zhu, & Tannenbaum, 2013). Such preferences are socially rational, because standard formulations of consequentialism require maximising the greater good even if this involves using, harming, and even killing innocent people. This consequentialist rejection of any constraints on the maximisation of welfare means that there is no place for rights, duties, and respect for individual persons: if by stealing your new laptop and selling it on the black market I could make a lot of money that I could donate to charities in the developing world to save children's lives, this is what I should do – regardless of whether I have previously made (potentially implicit) commitments not to steal from you. But expected adherence to such implicit commitments is critical when selecting a social partner for the purposes of cooperative exchange (e.g. friend, spouse, colleague). Indeed, we have argued that this tension between consequentialism and what we seek in social partners could, through mechanisms of partner choice (e.g. Baumard, André, & Sperber, 2013; Noë & Hammerstein, 1994), explain the prevalence of non-consequentialist moral intuitions (Everett et al., 2016). To the extent that people who make non-consequentialist moral judgments in sacrificial dilemmas are favored in a cooperation market - seemingly because these judgments signal a commitment to cooperation - deontological moral intuitions could therefore represent an evolutionarily prescribed prior that was selected for through partner choice mechanisms (Everett et al., 2016).

Although sacrificial moral dilemmas make good drama, these are not necessarily the most common conflict between consequentialist and deontological principles. As outlined in the two-dimensional model of utilitarian psychology (Kahane et al., 2018), consequentialist theories like utilitarianism involve more than just decisions about whether to sacrifice one to save a greater number (“*instrumental harm*”). At the core of utilitarianism is the idea of *impartial beneficence*, that we must impartially maximise the well-being of all sentient beings on the planet in such a way that “[e]ach is to count for one and none for more than one” (Bentham, 1789/1983), not privileging compatriots, family members, or ourselves over strangers – or even enemies. In general, people are attracted to impartiality, preferring fairness to unfairness (e.g. Fehr & Schmidt, 1999; Shaw, 2013; Tyler, 2000), and will choose equity over efficiency when these are in conflict, seemingly out of a desire to appear impartial (e.g. Choshen-Hillel, Shaw, & Caruso, 2015; Shaw, 2013). But there are also limits to preferences for impartiality, for example when balancing concerns of fairness and loyalty - as in the “whistleblowers dilemma” (Dungan, Waytz, & Young, 2014; Waytz, Dungan, & Young, 2013).

Critically, the strict consequentialist impartial standpoint departs substantially from common-sense morality, which posits *special obligations* to those with whom we have some kind of special relationship. Parents, for example, have certain duties and obligations to their children that are not shared by other people. These special obligations make it morally permissible (or even required) to save one's own child over, e.g., two strangers' children, and are incorporated into many forms of deontological ethics (e.g. Annis, 1987; Held, 2006; Kamm, 2007; Scanlon, 1998). Indeed – as we return to later - persistent philosophical criticisms of consequentialist theories centre on the fact that they fail to account for special obligations such as those found in friendship (e.g. Cocking & Oakley, 1995; Woodcock, 2009). Even if people prefer impartiality when deciding allocations between two family members, work colleagues, or strangers, we think it unlikely that people will prefer impartiality when deciding allocations between a single family member and a greater number of strangers. We know that when we read *The Iliad* we harshly judge Agamemnon for his consequentialist decision to sacrifice his daughter for the greater good. When reading Dicken's *Bleak House*, might we also think badly of the ‘telescopic philanthropist’ Mrs. Jellyby who spends most of her time setting up a charity for a far-off tribal community while ignoring the needs of her own family? We think the answer is yes.

Just as with instrumental harm, in the domain of impartial beneficence there is a deep conflict between what we seek in a social partner and the requirements of consequentialism. In the simplest terms, non-consequentialists should be preferred in the domain of instrumental harm because we do not want social partners who will harm us in order to maximise the greater good; non-consequentialists should be preferred in the domain of impartial beneficence because we want social partners who will help us even if it does not maximise the greater good.

Although we predict non-consequentialists would be preferred over consequentialists across both dimensions, it is also reasonable to assume that these preferences would be weaker when consequentialist preferences are expressed through endorsement of impartial beneficence than instrumental harm. Research on the omission bias shows that directly harming someone is judged as more morally wrong than failing to help or allowing harm to occur (Baron & Ritov, 1994; Ritov & Baron, 1990; Siegel, Crockett, & Dolan, 2017; Spranca, Minsk, & Baron, 1991), and psychologists have identified a general positive-negative psychological asymmetry whereby “bad is stronger than good” (Baumeister, Bratslavsky, Finkenauer, & Vohs, 2001), and negative information is seen as more diagnostic in impression formation and person perception (Skowronski & Carlston, 1989).

In this paper we report four studies in which we investigated social perceptions of non-consequentialist and consequentialist agents in both sacrificial dilemmas tapping endorsement of instrumental harm, and impartiality dilemmas tapping endorsement of impartial beneficence. As well as theoretically extending the conceptual space in which non-consequentialists might be preferred, we also investigate this across a much greater range of dependent measures than has previously been used. Specifically, we study partner preference by looking at two different economic games (the Trust Game and the Prisoner's Dilemma); several distinct dimensions along which the agent's character could be perceived (warmth; competence; morality); the different social roles in which the agent would be preferred (as a friend, a spouse, a boss, and as a political leader); and the different processes or motivations perceived to influence the agent's moral decision (reason vs. emotion; strategic considerations; altruistic motivations).

## 2 Study 1

### 2.1 Method

#### 2.1.1 Open science

We report all measures,^1^ manipulations, and exclusions, and all data, analysis code, and experiment materials are available for download at: https://osf.io/yuv2m/.

#### 2.1.2 Ethics statement

For all studies, relevant ethical guidelines were followed and the research was approved through University of Oxford's Central University Research Ethics Committee, with the reference number MS-IDREC-C1-2015-098.

#### 2.1.3 Participants

We recruited 201 participants via MTurk, and paid them $1.00 for their time. Participants were excluded from completing the survey if they had participated in related studies by us in the past, and were excluded from analysis if took the survey more than once (*N* = 4) or failed a simple comprehension check asking them to indicate the judgment their partner made in the dilemma (*N* = 5). This left us a final sample of 192 participants (98 female; *M*_age_ = 33, *SD* = 9.84). Our sample size was determined through an a priori power analysis (see supplementary methods for details) and a sensitivity power analysis for our main ANCOVA analysis, assuming an α of 0.05 and power of 0.80, indicated that the minimum effect size we had power to detect was a small-to-medium effect of *f* = 0.20.

#### 2.1.4 Design

This study had a two-group design (Agent Judgment: Non-Consequentialist vs. Consequentialist), where participants were asked to report their perceptions of a protagonist who made either a characteristically consequentialist or non-consequentialist decision in an impartiality dilemma. We included two different dilemmas to test the generality of any effects we observed and to demonstrate that findings were not specific to the one particular instantiation of the underlying impartiality dilemma. Given that the pattern of results was broadly the same^2^ across the two dilemmas, we collapsed responses across the two. Nonetheless, full results using each dilemma separately can be seen in the supplementary results.

#### 2.1.5 Procedure

Participants read one of two^3^ dilemmas in which the protagonist faced a decision whether to help a single member of her family, or instead to help a greater number of strangers. The first impartiality dilemma – “spending time” – was adapted from the “greater good” dilemmas developed by Kahane, Everett, Earp, Farias, and Savulescu (2015), and involved an engineer named Janet who had to decide whether to spend her weekend cheering up her lonely mother, or instead to help re-build houses for families who have lost theirs after flooding in the region. The second dilemma – “spending money” – involved a grandmother named Susan who had recently won a prize of $2000 and had to decide whether to donate this to the Against Malaria Foundation to provide mosquito nets to families in the developing world at risk of malaria, or to give this to her grandson to get his car fixed. In each of these dilemmas, the protagonist made either the characteristically deontological, non-consequentialist decision (i.e. to help the single family member) or the characteristically consequentialist one (i.e. to help the greater number of strangers), and gave a justification for doing so that either aligned more with the ethical dictates of consequentialist (“more happiness for more people”) or deontological (“duties she has”) theories (see the supplementary materials for the full text in Study 1; see Table 2 in the paper for the similar text used in Studies 2–4). After being introduced to the dilemma and what the protagonist chose to do, participants indicated what they thought she should have done, and rated aspects of the character of the protagonist in the dilemma.

#### 2.1.6 Measures

***Participant Moral Judgment*** in the moral dilemmas was measured using three questions. First, participants were asked to make a binary judgment about what they thought the protagonist in the dilemma should have done (e.g. “Volunteer to help build the houses” vs. “Spend the time cheering up her mother”). Second and third, participants were asked to indicate how morally wrong they thought it would be to make the consequentialist (e.g. volunteer) and non-consequentialist (e.g. spend the time with the mother) decisions on a 1–7 scale (*1* = *not at all morally wrong*; *7* = *extremely morally wrong*).

***Participant Consequentialist Tendencies*** were measured using the Oxford Utilitarianism Scale (OUS: Kahane et al., 2018), consisting of two subscales in which participants are asked to indicate how much they agree or disagree with 9 items. The first subscale - *Impartial Beneficence* (OUS-IB) - consists of 5 items (α = 0.70) that all tap endorsement of the impartial maximisation of the greater good even at the cost of personal self-sacrifice, such as “If the only way to save another person's life during an emergency is to sacrifice one's own leg, then one is morally required to make this sacrifice”. The second subscale - *Instrumental Harm* (OUS-IH) - consists of 4 items (α = 0.78) that tap a willingness to cause harm in order to bring about the greater good, including “It is morally right to harm an innocent person if harming them is a necessary means to helping several other innocent people”.

***Character Ratings*** were measured with seven questions in which participants rated on a 1–7 scale how they perceived the protagonist in the story to be in terms of how moral (*1* = *extremely immoral*/*bad*; *7* = *extremely moral*/*good*), trustworthy (*1* = *extremely untrustworthy*; *7* = *extremely trustworthy*), loyal (*1* = *extremely disloyal*; *7* = *extremely loyal*), reliable (*1* = *extremely unreliable*; *7* = *extremely reliable*), warm or cold (*1* = *extremely cold*; *7* = *extremely warm*), competent (*1* = *not at all competent*; *7* = *extremely competent*), and capable (*1* = *not at all capable*; *7* = *extremely capable*) they thought the protagonist in the story to be.

***Role Suitability*** were measured with four questions in which participants rated on 1–7 scale how good a partner they thought the protagonist in the story would be in four types of social roles: as a friend, as a spouse, as a boss, and as a political leader, specifically President of the United States (*1* = *an extremely bad X*; *7* = *an extremely good X*).

***Perceived Motivations*** of the protagonist's decision in the dilemma was measured through two items where participants indicated how much they thought the protagonist was driven by “altruistic, empathic motives”, and how much they thought the protagonist was driven by “strategic, reasoned motives” (*1* = *not at all*, *7* = *very much*).

#### 2.1.7 Analysis plan

Our primary measure of interest, like other recent studies (e.g. Hughes, 2017; Rom et al., 2017), was how the protagonist in the story was perceived depending on her decision, regardless of the judgment the participant themselves made about the dilemma. One source of variance that has not been controlled for in these previous studies, however, is participants' own moral judgments. Therefore, in this study (and both subsequent ones), we sought to control for participants' own moral judgments in the dilemmas by entering participant wrongness of the consequentialist action into an ANCOVA (consequentialist vs. non-consequentialist protagonist judgment with participant wrongness as a covariate). This allowed us to consider the interactive and main effects of interest while also controlling for any residual variance caused by participants' own judgments.^4^ Because the data was not normally distributed (for this study or the subsequent ones), we complemented the ANCOVA controlling for participant wrongness with a non-parametric Mann-Whitney *U* test (see supplementary results for results from Shapiro-Wilk tests of normality in each study). Means, standard deviations, *p*-values from the Mann-Whitney *U* tests, and effect sizes can be seen in Table 1.

**Table 1:** *M*s and *SD*s as a function of protagonist judgment in the dilemma (Study 1).

	Non-consq protagonist	Consequentialist protagonist	*p*-value	*d*	Prefer
Morality	5.91 (0.94)	5.93 (1.03)	.72	0.02	
Trustworthiness	6.02 (0.98)	5.60 (1.25)	.03	0.37	Non-C
Loyalty	6.55 (0.74)	4.64 (1.51)	<.001	1.61	Non-C
Reliability	5.99 (0.99)	5.24 (1.50)	<.001	0.59	Non-C
Warmth	6.27 (0.90)	5.28 (1.47)	<.001	0.81	Non-C
Competence	6.05 (1.01)	5.77 (1.19)	.11	0.25	
Capability	6.05 (0.98)	5.87 (1.11)	.31	0.17	
Suitability as a friend	6.10 (1.00)	5.05 (1.38)	<.001	0.87	Non-C
Suitability as a spouse	6.09 (0.96)	4.90 (1.45)	<.001	0.97	Non-C
Suitability as a boss	5.33 (1.20)	5.33 (1.32)	.88	0.00	
Suitability as a political leader	4.53 (1.42)	5.13 (1.47)	.002	0.42	Consq
Altruistic motives	5.53 (1.46)	5.49 (1.61)	.87	0.03	
Strategic motives	3.18 (1.73)	4.81 (1.86)	<.001	0.91	

For completeness, we also report in the supplementary materials a series of 2 × 2 ANOVAs in which we entered both participant consequentialist or non-consequentialist judgment and protagonist consequentialist or non-consequentialist decision. However, given that we were primarily interested in how the protagonist in the story was perceived overall depending on her decision, not how she was perceived differently by participants who themselves endorsed either option, in the interests of clarity and conciseness we have chosen to report these analyses in the supplementary materials.

### 2.2 Results

#### 2.2.1 Participant judgments

Overall, most participants endorsed the characteristically non-consequentialist option of helping the family member over the characteristically consequentialist option of helping the greater number of strangers (63%), but this was more pronounced in the spending money dilemma (81%) than the spending time dilemma (55%).While participants did not think that either action was morally wrong, participants who reported higher consequentialist tendencies thought it would be more wrong to help the family number over the greater number of strangers. In the interests of space, we report all further results on participant judgments in the supplementary materials.

#### 2.2.2 Character ratings

When the protagonist in the dilemma decided to help the family member over the greater number of strangers, they were perceived as significantly more trustworthy (*F*(1,189) = 7.23, *p* = .008, η_p_^2^ = 0.04; *U* = 3781, *p* = .026, *d* = 0.37), reliable (*F*(1,189) = 17.36, *p* < .001, η_p_^2^ = 0.08; *U* = 3341, *p* < .001, *d* = 0.59), loyal (*F*(1,189) = 128.23, *p* < .001, η_p_^2^ = 0.40; *U* = 1333, *p* < .001, *d* = 1.61), and warm (*F*(1,189) = 32.76, *p* < .001, η_p_^2^ = 0.15; *U* = 2730, *p* < .001, *d* = 0.81). There were, however, no differences between consequentialist and non-consequentialist agents in terms of perceived morality (*F*(1,189) = 0.00, *p* = .97, η_p_^2^ = 0.00; *U* = 4473, *p* = .72, *d* = 0.02), competence (*F*(1,189) = 3.35, *p* = .069, η_p_^2^ = 0.02; *U* = 4023, *p* = .11, *d* = 0.25), or capability (*F*(1,189) = 1.60, *p* = .21, η_p_^2^ = 0.00; *U* = 4235, *p* = .31, *d* = 0.17).

### 2.3 Role suitability

When the protagonist made the characteristically non-consequentialist decision to help the family member over the greater number of strangers they were expected to make a better friend (*F*(1,189) = 36.65, *p* < .001, η_p_^2^ = 0.16; *U* = 2519, *p* < .001, *d* = 0.87) and spouse (*F*(1,189) = 46.20, *p* < .001, η_p_^2^ = 0.20; *U* = 2417, *p* < .001, *d* = 0.97), but there was no difference in suitability as a boss (*F*(1,189) = 0.00, *p* = 1.00, η_p_^2^ = 0.00; *U* = 4545, *p* = .88, *d* = 0.00), and the protagonist was actually seen to make a better political leader if they made the characteristically consequentialist decision (*F*(1,189) = 8.62, *p* = .004, η_p_^2^ = 0.04; *U* = 3471, *p* = .002, *d* = 0.42).

#### 2.3.1 Perceived motivations

Participants thought the consequentialist protagonist was driven more by strategic, reasoned motives than the non-consequentialist protagonist, *F*(1,189) = 39.95, *p* < .001, η_p_^2^ = 0.17; *U* = 2409, *p* < .001, *d* = 0.91, but there was no difference between agents in perceptions of empathic, altruistic motives, *F*(1,189) = 0.07, *p* = .80, η_p_^2^ = 0.00; *U* = 4544, *p* = .87, *d* = 0.03.

### 2.4 Discussion

Results from Study 1 using impartiality dilemmas were less consistent than the unequivocal preference for the non-consequentialist in sacrificial dilemmas we have seen in previous work (Everett et al., 2016), but overall suggested that there may be a significant social cost of making a consequentialist judgment in the domain of impartial beneficence. Of the twelve measures, the non-consequentialist was preferred in six; there was no difference for five items; and for only one item was the consequentialist preferred. The non-consequentialist appeared to be favored primarily in the context of direct interpersonal relationships, being seen as more loyal, more reliable, more trustworthy, and warmer than a consequentialist actor, and being thought to make a better friend and spouse. In contrast, there was no difference for suitability as a boss, and the non-consequentialist was thought to make a worse political leader than the consequentialist.

Certain limitations of this study should be noted, however. First, Study 1 relied on character ratings of the protagonist and did not measure participants' actual behaviour. Although important work on this topic has been conducted without measuring behaviour (e.g. Hughes, 2017; Rom et al., 2017; Uhlmann et al., 2013), we think it is important to establish whether participants would actually be willing to ‘put their money where their mouth is’ and - like for the sacrificial dilemmas (Everett et al., 2016) - trust a non-consequentialist in the impartiality dilemmas with their money more than they do a consequentialist. Second, and relatedly, here we focused on ratings of hypothetical actors in moral dilemmas, but it is possible that results would be weaker when participants were thinking about (ostensibly) real other participants, not just judging a hypothetical protagonist.

## 3 Study 2

In Study 2 we looked again at perceptions of consequentialist agents endorsing impartial beneficence, this time adding an economic Trust Game to establish whether the partner preference has real behavioural consequences in a cooperation market, and turned back to examining a third-party judge, as in the majority of this work (Bostyn & Roets, 2017; Everett et al., 2016; Rom et al., 2017). We also wanted to replicate and extend previous findings that non-consequentialist judges in sacrificial dilemmas are rated as more moral and trustworthy and receive more transfers in an economic Trust Game. We sought to extend this by using two different sacrificial dilemmas, and by using a greater range of dependent measures than used in the original studies.

### 3.1 Method

#### 3.1.1 Open science

Our design, hypotheses, and analysis plan were all pre-registered at the Open Science Framework (https://osf.io/prm3a/). We report all measures,^5^ manipulations, and exclusions in this study. All data, analysis code, and experiment materials are available for download at: https://osf.io/bdev3/.

#### 3.1.2 Participants

In accordance with the pre-registration, we recruited 1000 participants via MTurk. Participants were excluded from completing the study if they had participated in related studies by us in the past, and were excluded from analysis if they did not complete the study in full (*N* = 6) or failed a simple comprehension check asking them to indicate the judgment their partner made in the dilemma (*N* = 41). This left us with a final sample of 953 participants (467 female; *M*_age_ = 34, *SD* = 10.44). Our sample size was determined through an a priori power analysis (see supplementary methods for details) and a sensitivity power analysis for our main 2 × 2 ANCOVA analysis, assuming an α of 0.05 and power of 0.80, indicated that the minimum effect size we had power to detect was a small effect of *f* = 0.09.

All participants were paid $1.80 for participating, in accordance with an hourly US minimum wage of $7.25 and the survey taking approximately 15 min. Participants were paid a bonus of up to $0.60 depending on their decision in the game. To determine bonuses, we recruited a separate group of participants to play as second movers, who answered one of the four moral dilemmas and then indicated what percentage of the money transferred to them they would return. We collected data until we had at least one participant in each dilemma who gave a consequentialist or non-consequentialist decision, and selected the first person who met that criteria (i.e. the first consequentialist in the spending money dilemma; the first non-consequentialist in the spending time dilemma). We then determined bonuses for participants within each condition by looking at what percentage the corresponding second mover said they would return and paid participants accordingly.

#### 3.1.3 Design

We had a 2 (Dilemma Type: Sacrificial vs. Impartiality × 2 (Agent Judgment: Consequentialist vs. Non-Consequentialist) between-subjects design, with two dilemmas in each category being used as variations of the experimental materials. Participants read one of four dilemmas and rated a partner (“agent”) who read the same dilemma and made either a consequentialist or non-consequentialist judgment and justification (see Table 2 for full text). All dilemmas involved a person called “Amy” who faces a dilemma with two decision options aligning more with consequentialism versus non-consequentialism. Two (‘footbridge’, ‘vaccine’) were sacrificial dilemmas (sacrificing one person to save a greater number of others), and two (‘spending money’, ‘spending time’) were impartiality dilemmas slightly adapted^6^ from Study 1 (helping a family member or greater number of strangers). Our primary interest was in the differences between the sacrificial and impartiality dilemmas, so we combined responses across the two sacrificial and two impartiality dilemmas.

**Table 2:** Reported judgments and justifications of the agent (“Person B”) in Studies 2–3.

		Non-consequentialist agent	Consequentialist agent
Sacrificial	Footbridge	“I think that Amy should not push the large man to save the five workers. I know that by doing this she could stop the trolley and save more lives, but I think that killing people is just wrong even if it has good consequences.”	“I think that Amy should push the large man to save the five workers. By doing this she could stop the trolley and save more lives, and I think that it is better to save many lives than just one”
	Vaccine	“I think that Amy should not inject the lab assistants with the substances to find out which is the vaccine. I know that by doing this she could find out which is the vaccine and therefore be able to save many more lives, but I think that killing people is just wrong, even if it has good consequences.”	“I think that Amy should inject the lab assistants with the substances to find out which is the vaccine. By doing this she could find out which is the vaccine and therefore be able to save many more lives, and I think that it is better to save many lives than just one”
Impartiality	Spending Money	“I think that Amy should give the $500 to her grandson so that he could get his car fixed. I know that by donating the money to charity Amy would have the chance to bring about more happiness for more people, but I also think that respecting the duties she has to her grandson is more important.”	“I think that Amy should give the $500 to the charity providing polio vaccinations in the developing world. I know that by donating the money to charity Amy would have the chance to bring about more happiness for more people, and I think this is more important than any duties she has to her grandson”
	Spending Time	“I think that Amy should spend the time with her mother instead of volunteering to build houses. I know that that by volunteering Amy would have the chance to bring about more happiness for more people, but I also think that respecting the duties she has to her mother is more important.”	“I think that Amy should spend the time volunteering to build houses instead of spending the time with her mother. I know that by volunteering Amy would have the chance to bring about more happiness for more people, and I think this is more important than any duties she has to her mother”

#### 3.1.4 Procedure

At the start of the study participants were introduced to one of the four dilemmas and asked to indicate which of two behaviours they thought Amy should do, how morally wrong it would be to perform the consequentialist action, and how much responsibility they perceived Amy to have to the people in the story. Next, participants were introduced to a Trust Game (TG; Berg, Dickhaut, & McCabe, 1995). The TG typically involves two participants: an investor (“Player A”), and a trustee (“Player B”). Player A is given some money and told that they may send a proportion (from zero to the full amount) of this money to the trustee, and that the experimenter will multiply the money sent by some amount. Once Player B receives the money, they are told that they may send back a portion of it to the investor, again ranging from zero to the full amount. In our study, participants (who always played the role of Person A) were able to allocate between $0.00 and $0.30, and any money they sent to Person B was doubled. Participants were then required to successfully complete three comprehension questions about the structure of the game to move to the next page. Participants were then informed that they were completing this task playing as Person A and that we had already conducted a first-wave of data collection in which we recruited people to play as Person B (the second player, who returns money back to the first player) and had them indicate, for all the possible amounts they could receive from Person A, how much they wanted to return. Participants learned that they would be told how Person B responded in the moral dilemma they answered at the start of the survey, and then their own task was to decide how much - if anything - they wanted to transfer to Person B, with their eventual bonus being dependent on how much the participant decided to transfer, and how much Person B decided to return back. After receiving the information about the TG and their role in the study, participants entered the main stage of the study where they were randomly assigned to play with an agent (Person B) who either made a consequentialist or non-consequentialist choice and justification in the moral dilemma participants read at the start of the study (see Table 2). Participants were told of the agent's judgment and reasoning and completed a comprehension check to confirm they understood what judgment the agent/Person B chose (41 participants answered this incorrectly and were excluded from data analysis). Participants then completed the TG and the other dependent measures (see below).

#### 3.1.5 Measures

***Participant Moral Judgment*** in the moral dilemma was measured in two ways: first, as a binary judgment in which participants indicated what they thought Amy should do in the moral dilemma (e.g. “I think that Amy should spend the time with her mother” vs. “I think that Amy should spend the time volunteering”); and second, as a continuous measure on an eleven-point scale of how wrong participants thought it would be to perform the consequentialist action instead of the non-consequentialist one (−*5* = *absolutely morally wrong*/*forbidden*; *0* = *neither right nor wrong*; *5* = *absolutely morally required*/*should always be done*).

***Partner Preference*** was measured by a single question in which participants were asked to indicate what kind of partner they would have preferred to play the TG with if they had a choice: someone who made a consequentialist or non-consequentialist judgment.

***Role Suitability***, like in Study 1, was measured with four questions in which participants rated on 1–7 scale how good a partner they thought the protagonist in the story would be in four types of social roles: as a friend, as a spouse, as a boss, and as political leader, i.e. President of the United States (*1* = *an extremely bad X*; *7* = *an extremely good X*).

***Character Ratings*** were measured with seven questions in which participants rated on 1–7 scale how moral, trustworthy, loyal, warm or cold, sociable, competent, and capable they thought the agent to be. Ratings of how sociable (*1* = *not at all sociable*; *7* = *extremely sociable*) and warm or cold (*1* = *extremely cold*; *7* = *extremely warm*) participants thought the agent was were combined into an overall score of warmth (α = 0.76); and ratings of how competent (*1* = *not at all competent*; *7* = *extremely competent*) and capable (*1* = *not at all capable*; *7* = *extremely capable*) participants thought the agent was were combined into an overall score of competence (α = 0.88). Ratings of how moral (*1* = *extremely immoral*/*bad*; *7* = *extremely moral*/*good*) and trustworthy (*1* = *extremely untrustworthy*; *7* = *extremely trustworthy*) participants thought the agent were combined into an overall score of morality (α = 0.85). We analyzed loyalty separately because in the impartiality dilemmas but not the sacrificial ones the non-consequentialist action strongly involved loyalty (to one's parents). We created these composite scores both in the interests of space and because, given they are intended to measure the same construct, we expected – and found - no differences between the results for each. Nonetheless, full results using the individual items can be seen in the supplementary results.

#### 3.1.6 Analysis plan

As outlined in the pre-registration, our primary analysis of interest was on the main effects of agent consequentialist or non-consequentialist judgment in both the sacrificial and impartiality dilemmas, and whether there was an interaction effect between them such that the effect was stronger for one type of dilemma. Across Studies 2–4, we used a 2 × 2 ANCOVA (sacrificial vs. impartiality dilemma; consequentialist vs. non-consequentialist agent judgment; participant ratings of wrongness of consequentialist action as a covariate) to look at the interactive effect of dilemma type and agent judgment and explore our main effects while controlling for any variance caused by participants' own judgments. Because the data was again not normally distributed we complemented this ANCOVA with non-parametric Mann-Whitney *U* tests (see supplementary results for results from Shapiro-Wilk tests of normality in each study). All *M*s and *SD*s as a function of dilemma type and agent judgment, along with the *p*-values from a Mann-Whitney *U* test and effect size of the difference between a consequentialist and non-consequentialist agent for each type of dilemma can be seen in Table 3.

**Table 3:** *M*s, *SD*s, *p*-values and effects sizes in Study 2 as a function of dilemma type and agent judgment.

	Sacrificial dilemmas					Impartiality dilemmas				
	Non-conseq.	Consq.	*p*-value	*d*	Prefer	Non-conseq.	Consq.	*p*-value	*d*	Prefer
Transfer amounts	20.10 (11.76)	17.23 (11.87)	.006	0.23	Non-C	18.46 (11.97)	18.59 (11.93)	.98	0.01	–
Predicted returns	37.53 (24.60)	31.15 (24.04)	.005	0.26	Non-C	32.97 (23.46)	37.57 (25.86)	.04	−0.19	Consq
Preferred partner in TG	70%	30%	<.001	N/A	Non-C	53%	47%	.16	N/A	–
Morality	5.55 (1.01)	4.37 (1.22)	<.001	1.04	Non-C	5.10 (1.05)	5.04 (1.11)	.56	0.05	–
Warmth	5.57 (0.97)	4.52 (1.16)	<.001	0.98	Non-C	5.11 (1.07)	5.14 (1.14)	.68	−0.03	–
Competence	5.35 (1.15)	5.02 (1.19)	.002	0.29	Non-C	5.35 (1.14)	5.28 (1.08)	.34	0.06	–
Loyalty	5.54 (1.15)	4.19 (1.51)	<.001	1.01	Non-C	5.95 (1.04)	4.50 (1.44)	<.001	1.15	Non-C
Suitability as a friend	5.59 (1.08)	4.31 (1.45)	<.001	1.00	Non-C	5.39 (1.18)	4.78 (1.27)	<.001	0.50	Non-C
Suitability as a spouse	5.28 (1.17)	4.04 (1.44)	<.001	0.95	Non-C	5.25 (1.24)	4.55 (1.36)	<.001	0.54	Non-C
Suitability as a boss	4.98 (1.45)	4.13 (1.55)	<.001	0.57	Non-C	4.75 (1.44)	4.70 (1.37)	.49	0.04	–
Suitability as a political leader	4.33 (1.58)	3.97 (1.75)	.04	0.22	Non-C	4.08 (1.42)	4.46 (1.51)	.003	−0.26	Consq

In accordance with the pre-registration, in the supplementary materials we also report (1) main effects of agent judgment for each dilemma separately, and (2) results from a 2 × 2 × 2 ANOVA in which we entered participant moral judgment as a fixed factor (instead of a covariate). As can be seen in the supplementary materials, results were consistent across the two dilemmas in each category, and we only found significant 3-way interactions in four of the eleven DVs (indicating that non-consequentialist and consequentialist participants responded significantly differently in the different dilemmas).

### 3.2 Results

#### 3.2.1 Participant judgments

The majority of participants endorsed the non-consequentialist option in the sacrificial dilemmas (69%), rejecting the sacrifice of one to save the lives of a greater number. This was the same for the footbridge (69%) and vaccine (69%) variants. Similarly, most participants endorsed the non-consequentialist option in the impartiality dilemmas (70%), endorsing helping a family member over impartially helping a greater number. This was the same for both dilemmas but was more pronounced in the spending money (83%) than the spending time variant (55%). Higher scores on the Impartial Beneficence subscale of the OUS predicted lower wrongness judgments of the consequentialist action in the impartiality dilemmas (*r* = 0.22 *p* < .001), and higher scores on the Instrumental Harm subscale predicted lower wrongness judgments of the consequentialist action in the sacrificial dilemmas (*r* = 0.62, *p* < .001) (see supplementary materials for further results looking at participant judgment).

#### 3.2.2 Character ratings

**Fig. 1:**
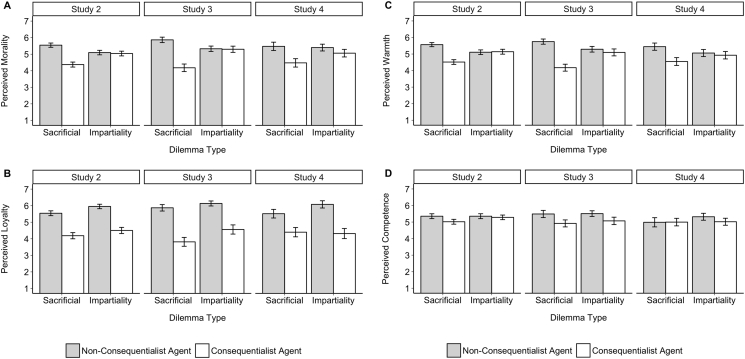
Character ratings in Studies 2–4 as a function of agent judgment and dilemma type. Results show that in the sacrificial dilemmas, the non-consequentialist was consistently rated as higher in morality (1A), loyalty (1B), warmth (1C), and competence (1D). In the impartiality dilemmas, the non-consequentialist was seen as more loyal but was not consistently rated as more moral, warm, or competent. Error bars represent 95% confidence intervals.

We first looked at character ratings (see Fig. 1). For perceived morality, the ANCOVA revealed the predicted main effect of agent judgment, *F*(1,948) = 74.69, *p* < .001, η_p_^2^ = 0.07; *U* = 147,829, *p* < .001, *d* = 0.56, and a significant interaction of dilemma type and agent judgment on how moral participants perceived the agent to be, *F*(1,948) = 61.30, *p* < .001, η_p_^2^ = 0.06. While a non-consequentialist was seen as more moral than the consequentialist in the sacrificial dilemmas, *F*(1,489) = 138.84, *p* < .001, η_p_^2^ = 0.22; *U* = 46,702, *p* < .001, *d* = 1.04, there was no difference in perceived morality of non-consequentialist vs. consequentialist agents in the impartiality dilemmas, *F*(1,458) = 0.33, *p* = .56, η_p_^2^ = 0.00; *U* = 27,382, *p* = .56, *d* = 0.05.

For perceived warmth, the ANCOVA revealed the predicted main effect of agent judgment, *F*(1,948) = 52.71, *p* < .001, η_p_^2^ = 0.05; *U* = 143,077, *p* < .001, *d* = 0.47, and a significant interaction of dilemma type and agent judgment on how warm and sociable participants perceived the agent to be, *F*(1,948) = 59.01, *p* < .001, η_p_^2^ = 0.06. While a non-consequentialist was seen as warmer than the consequentialist in the sacrificial dilemmas, *F*(1,489) = 119.95, *p* < .001, η_p_^2^ = 0.20; *U* = 45,840, *p* < .001, *d* = 0.98, there was no difference in the impartiality dilemmas, *F*(1,458) = 0.08, *p* = .77, η_p_^2^ = 0.00; *U* = 25,980, *p* = .68, *d* = 0.03.

For perceived competence, the ANCOVA showed the predicted main effect of agent judgment, *F*(1,948) = 7.53, *p* = .001, η_p_^2^ = 0.01; *U* = 125,545, *p* = .004, *d* = 0.18, and a significant interaction effect of agent judgment and dilemma judgment, *F*(1,948) = 3.24, *p* = .006, η_p_^2^ = 0.00. While a non-consequentialist was seen as more competent than the consequentialist in the sacrificial dilemmas, *F*(1,489) = 10.10, *p* = .002, η_p_^2^ = 0.02; *U* = 34,981, *p* = .002, *d* = 0.29, there was no difference in the impartiality dilemmas, *F*(1,458) = 0.46, *p* = .50, η_p_^2^ = 0.00; *U* = 27,898, *p* = .34, *d* = 0.06.

Finally, we turned to the single item of perceived loyalty. The ANCOVA revealed the predicted main effect of agent judgment, *F*(1,948) = 283.97, *p* < .001, η_p_^2^ = 0.23; *U* = 174,248, *p* < .001, *d* = 1.07, and there was no significant interaction of dilemma type and agent judgment, *F*(1,948) = 0.27, *p* = .61, η_p_^2^ = 0.00. For both dilemma types, an agent who made a non-consequentialist judgment was seen as more loyal than one who made a consequentialist judgment. This was the case both in the sacrificial dilemmas *F*(1,489) = 128.14, *p* < .001, η_p_^2^ = 0.21; *U* = 45,614, *p* < .001, *d* = 1.01, and the impartiality dilemmas, *F*(1,458) = 159.22, *p* < .001, η_p_^2^ = 0.26; *U* = 41,528, *p* < .001, *d* = 1.15.

Overall, then, across all the character ratings there were significant main effects such that the non-consequentialist was seen as more moral, warmer, more competent, and more loyal (see Fig. 1). With the exception of loyalty, however, these results were limited to the sacrificial dilemmas. In the impartiality dilemmas, the consequentialist was not seen as less moral, warm, or competent than the non-consequentialist.

#### 3.2.3 Trust Game

**Fig. 2:**
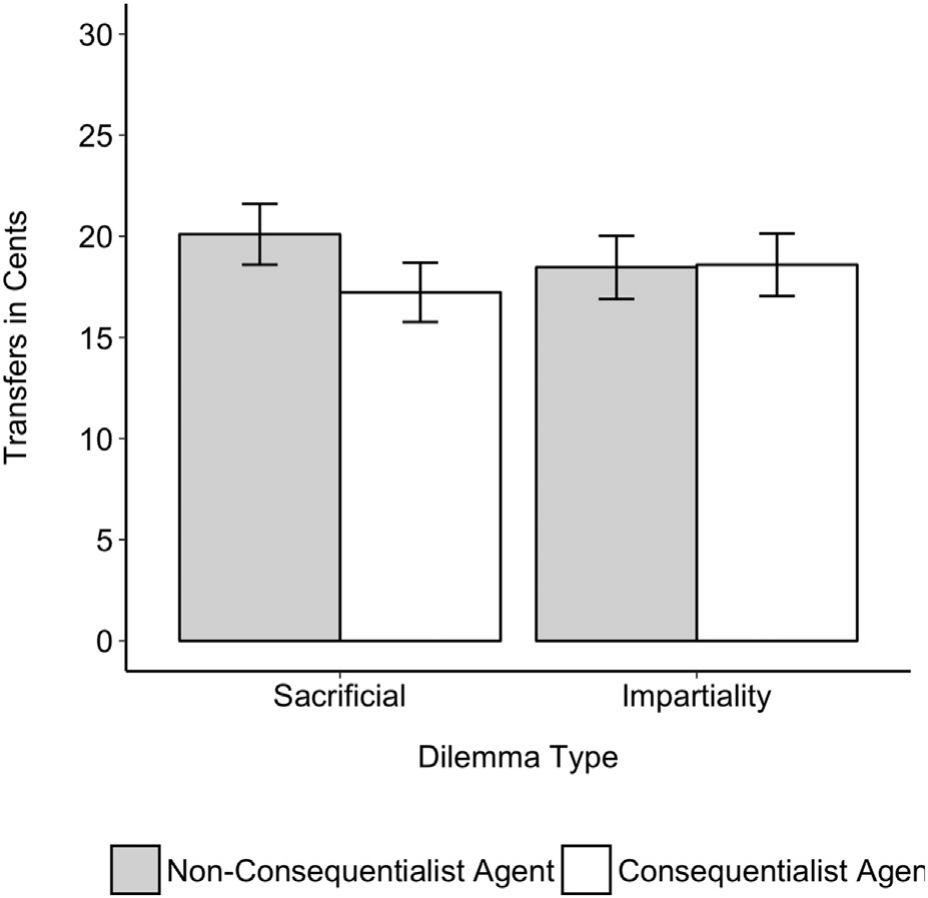
Transfers in a Trust Game as a function of agent judgment and dilemma type. Results show that in the sacrificial dilemmas participants transferred more to a non-consequentialist than a consequentialist, but there was no difference in the impartiality dilemmas. Error bars represent 95% confidence intervals.

An ANCOVA revealed a marginally significant interaction of dilemma type and agent judgment on transfer amounts, *F*(1,948) = 3.80, *p* = .052, η_p_^2^ = 0.00, and though there was no main effect of agent judgment when using the ANCOVA controlling for participant wrongness, *F*(1,948) = 3.18, *p* = .075, η_p_^2^ = 0.00, there was a significant main effect in the non-parametric test (because the data was not normally distributed), *U* = 121,638, *p* = .043, *d* = 0.12. Looking at simple effects, we found that participants transferred significantly more to a non-consequentialist agent than a consequentialist agent in the sacrificial dilemmas, *F*(1,489) = 7.22, *p* = .007, η_p_^2^ = 0.001; *U* = 34,431, *p* = .005, *d* = 0.24, but there was no difference in the impartiality dilemmas, *F*(1,458) = 0.01, *p* = .91, η_p_^2^ = 0.00; *U* = 26,593, *p* = .098, *d* = 0.01 (see Fig. 2). As an exploratory analysis, we looked at behaviour in the TG for each dilemma separately and found that our overall non-significant ANCOVA effect was driven by non-consequentialists being preferred over consequentialists only in the vaccine sacrificial dilemma, and not the footbridge (see supplementary results). While this might be surprising given our robust effects found in previous work, we think it likely this is driven by MTurk participants being overly familiar with the footbridge dilemma.

For predicted returns, surprisingly an ANCOVA revealed no significant interaction of dilemma type and agent judgment on how much participants expected their partner to return, *F*(1,948) = 12.02, *p* = .57, η_p_^2^ = 0.01, and no main effect of agent judgment, *F*(1,948) = 0.33, *p* = .57, η_p_^2^ = 0.00; *U* = 111,551, *p* = .49, *d* = 0.04. While the interaction was non-significant, simple effects revealed that a non-consequentialist agent was predicted to return more than the consequentialist in the sacrificial dilemmas, *F*(1,489) = 8.53, *p* = .004, η_p_^2^ = 0.002; *U* = 34,822, *p* = .002, *d* = 0.26, but the consequentialist was predicted to return more than the non-consequentialist in the impartiality dilemmas, *F*(1,458) = 3.99, *p* = .046, η_p_^2^ = 0.001; *U* = 23,683, *p* = .036, *d* = −0.19.

#### 3.2.4 Partner preference

Next, we looked at which type of person the participant would have liked to play the TG with, if they'd had a choice: the consequentialist or non-consequentialist. A logistic regression revealed a significant effect of dilemma type, *B* = −0.70, *SE* = 0.14, *Z* = −5.16, *p* < .001, with chi-square analyses revealing that while most participants (70%) preferred the non-consequentialist in the sacrificial dilemmas, *x*^2^(1) = 75.71, *p* < .001, there was no difference in preference for a non-consequentialist (53%) or consequentialist (47%) in the impartial dilemmas, *x*^2^(1) = 1.95, *p* = .16.

#### 3.2.5 Role suitability

**Fig. 3:**
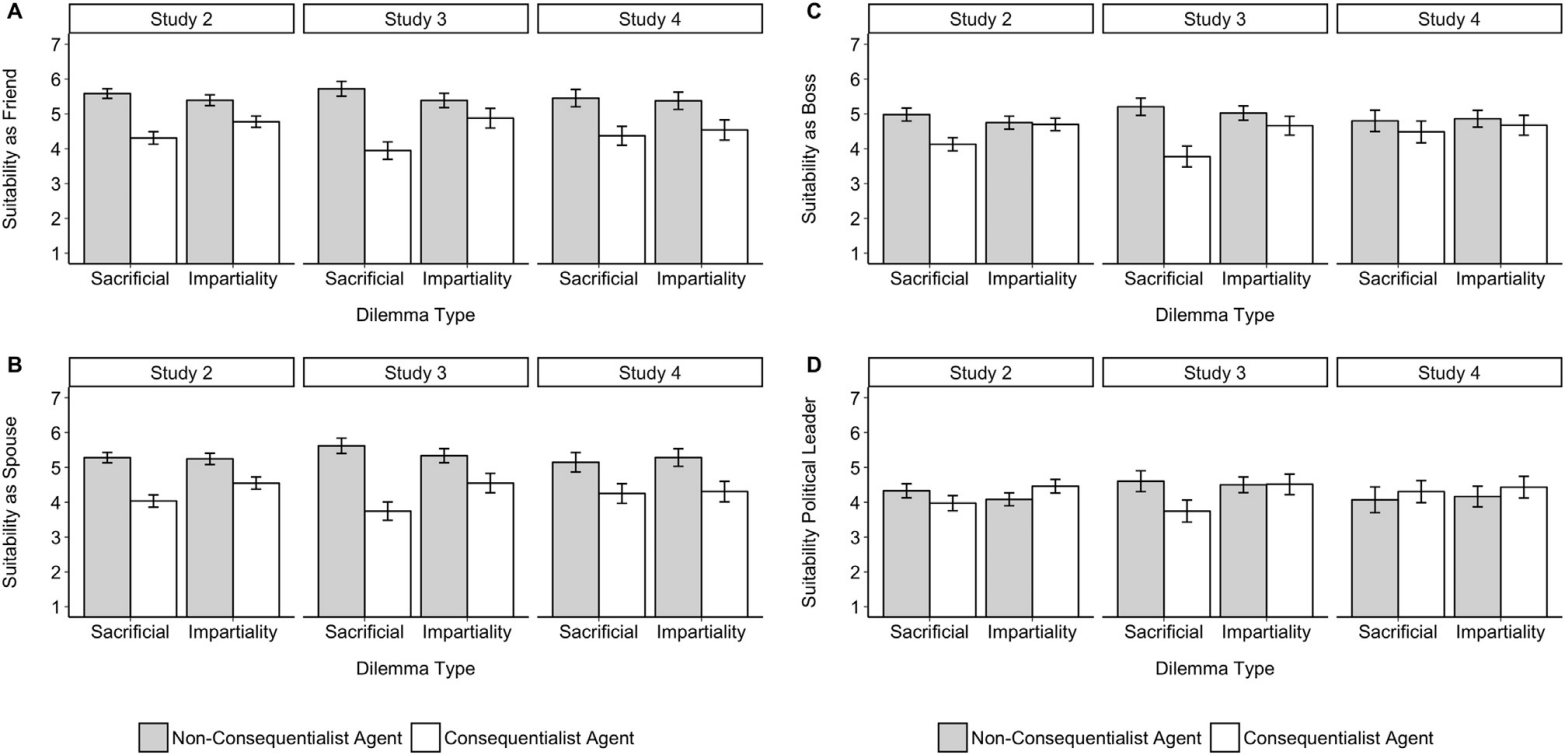
Role suitability in Studies 2–4 as a function of agent judgment and dilemma type. Results show that in the sacrificial dilemmas, the non-consequentialist was preferred for all four social roles. In the impartiality dilemmas, the non-consequentialist was consistently preferred as a friend (3A) and spouse (3B), but not a boss (3C) or political leader (3D). Error bars represent 95% confidence intervals.

Next, we looked at perceived suitability for different roles (see Fig. 3). Considering perceived suitability as a friend, an ANCOVA revealed the predicted main effect of agent judgment, *F*(1,948) = 137.60, *p* < .001, η_p_^2^ = 0.13; *U* = 68,383, *p* < .001, *d* = 0.76, along with a significant interaction of dilemma type and agent judgment on how good a friend participants thought the agent would be, *F*(1,948) = 16.78, *p* < .001, η_p_^2^ = 0.02. In both dilemma types, when the agent made a non-consequentialist judgment they were expected to make a better friend, but this was stronger for the sacrificial dilemmas, *F*(1,489) = 123.59, *p* < .001, η_p_^2^ = 0.20; *U* = 45,524, *p* < .001, *d* = 1.00, than for the impartiality dilemmas, *F*(1,458) = 29.66, *p* < .001, η_p_^2^ = 0.06; *U* = 33,921, *p* < .001, *d* = 0.50.

For perceived suitability as a spouse, an ANCOVA revealed the predicted main effect of agent judgment, *F*(1,948) = 133.71, *p* < .001, η_p_^2^ = 0.12; *U* = 69,310, *p* < .001, *d* = 0.75, along with a significant interaction of dilemma type and agent judgment on how good a friend participants thought the agent would be, *F*(1,948) = 10.84, *p* = .001, η_p_^2^ = 0.01. In both dilemmas, when the agent made a non-consequentialist judgment they were expected to make a better spouse, but this was stronger for the sacrificial dilemmas, *F*(1,489) = 112.39, *p* < .001, η_p_^2^ = 0.19; *U* = 44,716, *p* < .001, *d* = 0.95, than for the impartiality dilemmas, *F*(1,458) = 33.59, *p* < .001, η_p_^2^ = 0.07; *U* = 34,319, *p* < .001, *d* = 0.54.

For suitability as a boss, an ANCOVA revealed the predicted main effect of agent judgment, *F*(1,948) = 22.95, *p* < .001, η_p_^2^ = 0.02; *U* = 133,232, *p* < .001, *d* = 0.32 and a significant interaction of dilemma type and agent judgment on how good a boss participants thought the agent would be, *F*(1,948) = 18.12, *p* < .001, η_p_^2^ = 0.02. When the agent made a non-consequentialist judgment in the sacrificial dilemmas they were expected to make a better boss, *F*(1,489) = 39.98, *p* < .001, η_p_^2^ = 0.08; *U* = 39,299, *p* < .001, *d* = 0.57, but there was no difference for the impartiality dilemmas *F*(1,458) = 0.15, *p* = .70, η_p_^2^ = 0.00; *U* = 27,519, *p* = .49, *d* = 0.04.

For suitability as a political leader, an ANCOVA revealed a significant interaction of dilemma type and agent judgment on how good a political leader participants thought the agent would be, *F*(1,948) = 12.81, *p* < .001, η_p_^2^ = 0.01, but no main effect of agent judgment, *F*(1,948) = 0.01, *p* = .92, η_p_^2^ = 0.00; *U* = 111,573, *p* = .65, *d* = 0.00. Looking at simple effects, in the sacrificial dilemmas a non-consequentialist agent was expected to make a better political leader, *F*(1,489) = 5.54, *p* = .019, η_p_^2^ = 0.01; *U* = 33,389, *p* = .040, *d* = 0.21, but in the impartiality dilemmas it was a consequentialist agent who was expected to make a better political leader, *F*(1,458) = 7.55, *p* = .006, η_p_^2^ = 0.002; *U* = 22,423, *p* = .003, *d* = −0.26.

Overall, results showed that a non-consequentialist agent in the sacrificial dilemmas was seen to make a better friend, boss, spouse, and political leader than the agent who made a consequentialist judgment (see Fig. 3). In the impartiality dilemmas, a non-consequentialist was also seen as making a better friend and spouse, while there was no difference for suitability as a boss, and the consequentialist had the edge for consideration as a political leader.

### 3.3 Discussion

In Study 2 we had two key aims: first, to investigate how participants perceived and interacted with someone who made a consequentialist or non-consequentialist judgment in impartiality dilemmas; and second, to replicate previous work demonstrating partner preference in sacrificial dilemmas in a pre-registered study with new measures and a different dilemma.

In the sacrificial dilemmas we successfully replicated and extended previous findings, showing that people who make non-consequentialist judgments in sacrificial dilemmas are perceived more positively than those who make a consequentialist judgment. Non-consequentialists received more transfers in a Trust Game, were seen as more moral and trustworthy, and when given a choice participants indicated they would rather play with a non-consequentialist than a consequentialist. Moreover, we found that this preference for non-consequentialists extended to character ratings of warmth, competence, and loyalty, and that non-consequentialists were seen to make a better friend, a better boss, a better spouse, and a better political leader. In every single dependent measure, a person making a non-consequentialist judgment in a sacrificial dilemma was preferred.

In the impartiality dilemmas, the picture was much less consistent. The consequentialist in the impartial dilemmas was preferred on two measures: they were expected to return more in the TG, and they were thought to make a better political leader. The non-consequentialist was preferred on three measures: they were expected to be more loyal and make a better friend and spouse. For most measures, however, there were no differences between the consequentialist and non-consequentialist: they were seen as equally moral, warm, and competent; they were thought to make an equally good boss. While impartial consequentialists were expected to make a better political leader, they were thought to make a worse friend and spouse; and while they were expected to return more in a TG, this did not translate into actual increased transfers by participants, nor greater selection of the consequentialist for a future TG.

One potential limitation is that while 83% of participants endorsed the non-consequentialist judgment in the spending money variant, only 55% of participants did so in the spending time variant. Moreover, in this study – and in contrast to Study 1 – we did not find the expected negative correlation between OUS impartial beneficence scores and perceived wrongness of the consequentialist action in the spending time dilemma. This suggests that in this study participants themselves did not actually perceive the spending time dilemma to be one in which one action is consequentialist, and if this is the case, one must be careful about inferring too much about how people think about “consequentialist” judgers in the same dilemma (see supplementary results for analyses looking only at the spending money variant).

## 4 Study 3

In Study 3, we sought to replicate and extend the results in Study 2 using a streamlined set of moral judgment scenarios and a Prisoner's Dilemma (PD: Axelrod, 1980; Rapoport & Chammah, 1965) instead of a Trust Game. The PD is a 2-person social dilemma that exemplifies the tension that exists between individual rationality and group rationality: each individual receives a higher payoff for defecting from what is in the collective interest than for cooperating, but all individuals are better off if they all cooperate than if they all defect (Dawes, 1980; Hardin, 1968, Hardin, 1998). Cooperation in the PD requires both the goal of cooperation and the expectation that others will cooperate (Pruitt & Kimmel, 1977), or in other terms, a social preference to cooperate and a belief that others will too (Everett et al., 2015a, Everett et al., 2015b). Using the PD allowed us to assess not only the expectation that participants might have that consequentialists are less likely to cooperate than non-consequentialists, but also to study whether participants who themselves made a consequentialist or non-consequentialist judgment would be more likely to cooperate.

### 4.1 Method

#### 4.1.1 Open science

Our design, hypotheses, and analysis plan were all pre-registered at the Open Science Framework (https://osf.io/dfz2j/) as part of the Pre-Registration Challenge. We report all measures, manipulations, and exclusions in this study. All data, analysis code, and experiment materials are available for download at: https://osf.io/v6z53/.

#### 4.1.2 Participants

In accordance with the pre-registration, 498 participants completed the survey online via MTurk. Participants were excluded from completing the survey if they had participated in related studies by us in the past, and were excluded from analysis if they completed the survey more than once (*N* = 5), or failed a simple comprehension check asking them to indicate the decision their partner made in the dilemma (*N* = 12), leaving us with a final sample of 485 participants (250 female; *M*_age_ = 35, *SD* = 11.13). Our sample size was determined through an a priori power analysis (see supplementary methods for details) and a sensitivity power analysis for our main 2 × 2 ANCOVA analysis, assuming an α of 0.05 and power of 0.80, indicated that the minimum effect size we had power to detect was a small effect of *f* = 0.13. All participants were paid $1.80 for participating and were again paid bonuses depending on their decision in the game. To calculate bonuses, we selected the decision (to cooperate or defect) of the first participant who met the criteria for each condition (e.g. who had the sacrificial dilemma, gave a consequentialist decision themselves, and were playing with a non-consequentialist agent) and then matched participants accordingly (see supplementary file for more information).

#### 4.1.3 Design

This study had a 2 (Dilemma Type: Sacrificial vs. Impartiality) × 2 (Agent Judgment: Non-Consequentialist × Consequentialist) between-subjects experimental design. The study was identical to Study 2, except we used two dilemmas (the vaccine for the sacrificial dilemma, and the spending money for the impartiality dilemma) instead of four, and used a PD instead of a TG.

#### 4.1.4 Procedure

Like in Study 2, participants were first introduced to the moral dilemma and asked to make their own judgments. Next, participants were introduced to the PD and received extensive training about the structure of the game based on the procedure of Everett, Ingbretsen, Cushman, and Cikara (2017) (see materials on OSF for full instructions: https://osf.io/v6z53). Participants were required to complete four comprehension questions about the payoffs to each player depending on both player's choices in an example matrix, and they had to answer these correctly in order to move to the next page. After completing this, participants entered the main part of the study where they were told what decision their partner made in the moral dilemma, and made their choice either to cooperate (X) or defect (Y) in the main PD. For the main PD, if both players cooperated they received 3 points each; if both defected they received 1 point each; and if one defected and one cooperated, the defector received 5 points and the cooperator received 0 points.

#### 4.1.5 Analysis plan

The analysis plan for this study was identical to that in Study 2. See Table 4 for *M*s and *SD*s as a function of dilemma type and agent judgment, as well as *p*-values from a Mann-Whitney *U* test, and effect sizes. In accordance with the pre-registration, for the supplementary materials we again ran analyses looking at a 2 × 2 × 2 ANOVA in which we entered participant moral judgment as a fixed factor (instead of a covariate). There were, however, no significant 3-way interactions, further justifying our focus on the ANCOVA.

**Table 4:** *M*s, *SD*s, *p*-values and effects sizes in Study 3 as a function of dilemma type and agent judgment.

	Sacrificial dilemmas					Impartiality dilemmas				
	Non-conseq.	Consq.	*p*-value	*d*	Prefer	Non-conseq.	Consq.	*p*-value	*d*	Prefer
Morality	5.87 (0.89)	4.18 (1.33)	<.001	1.49	Non-C	5.33 (0.93)	5.30 (0.98)	.53	0.04	–
Warmth	5.75 (0.85)	4.18 (1.23)	<.001	1.49	Non-C	5.29 (0.98)	5.10 (1.09)	.08	0.19	–
Competence	5.49 (1.18)	4.92 (1.24)	<.001	0.47	Non-C	5.51 (0.98)	5.07 (1.16)	.004	0.42	Non-C
Loyalty	5.87 (1.06)	3.81 (1.57)	<.001	1.54	Non-C	6.13 (0.86)	4.56 (1.45)	<.001	1.33	Non-C
Cooperation in PD	73%	57%	.009	N/A	Non-C	71%	72%	.88	N/A	–
Partner Preference	68%	22%	<.001	N/A	Non-C	62%	28%	<.001	N/A	Non-C
Suitability as a friend	5.72 (1.15)	3.95 (1.46)	<.001	1.35	Non-C	5.39 (1.17)	4.88 (1.47)	.01	0.39	Non-C
Suitability as a spouse	5.62 (1.20)	3.75 (1.54)	<.001	1.35	Non-C	5.34 (1.16)	4.55 (1.46)	<.001	0.61	Non-C
Suitability as a boss	5.21 (1.36)	3.78 (1.75)	<.001	0.91	Non-C	5.02 (1.18)	4.66 (1.41)	.03	0.28	Non-C
Suitability as a political leader	4.60 (1.63)	3.75 (1.85)	<.001	0.49	Non-C	4.50 (1.28)	4.51 (1.53)	.76	0.00	–

### 4.2 Results

#### 4.2.1 Participant judgments

The majority of participants endorsed the non-consequentialist option in the sacrificial dilemma (68%), rejecting the sacrifice of one to save the lives of a greater number. Similarly, most participants endorsed the non-consequentialist option in the impartiality dilemma (79%), endorsing helping a family member over impartially helping a greater number. As for the previous two studies, further results looking at participant judgment can be found in the supplementary materials.

#### 4.2.2 Character ratings

We first looked at character ratings (see Fig. 1). For the two items measuring perceived morality (α = 0.89), the ANCOVA revealed the predicted main effect of agent judgment, *F*(1,480) = 84.19, *p* < .001, η_p_^2^ = 0.15; *U* = 41,514, *p* < .001, *d* = 0.79, and a significant interaction of dilemma type and agent judgment on how moral participants perceived the agent to be, *F*(1,480) = 72.68, *p* < .001, η_p_^2^ = 0.13. While a non-consequentialist was seen as more moral than the consequentialist in the sacrificial dilemma, *F*(1,247) = 142.20, *p* < .001, η_p_^2^ = 0.37; *U* = 13,230, *p* < .001, *d* = 1.47, there was no difference in the impartiality dilemma, *F*(1,232) = 0.11, *p* = .74, η_p_^2^ = 0.00; *U* = 7080, *p* = .65, *d* = 0.03.

For perceived the two items measuring warmth (α = 0.74), the ANCOVA revealed the predicted main effect of agent judgment, *F*(1,480) = 87.88, *p* < .001, η_p_^2^ = 0.15; *U* = 42,494, *p* < .001, *d* = 0.83, and a significant interaction of dilemma type and agent judgment on how warm and sociable participants perceived the agent to be, *F*(1,480) = 50.61, *p* < .001, η_p_^2^ = 0.10. While a non-consequentialist was seen as warmer than the consequentialist in the sacrificial dilemmas, *F*(1,247) = 140.09, *p* < .001, η_p_^2^ = 0.36; *U* = 13,218, *p* < .001, *d* = 1.47, there was no difference in the impartiality dilemmas, *F*(1,232) = 2.03, *p* = .16, η_p_^2^ = 0.01; *U* = 7700, *p* = .097, *d* = 0.18.

For the two items measuring perceived competence (α = 0.89), the ANCOVA showed the predicted main effect of agent judgment, *F*(1,480) = 23.96, *p* < .001, η_p_^2^ = 0.05; *U* = 36,630, *p* < .001, *d* = 0.45, but no interaction between agent judgment and dilemma type, *F*(1,480) = 0.28, *p* = .60, η_p_^2^ = 0.00. The non-consequentialist was seen as more competent in both the sacrificial, *F*(1,247) = 13.57, *p* < .001, η_p_^2^ = 0.05; *U* = 9895, *p* < .001, *d* = 0.47, and impartiality dilemmas, *F*(1,232) = 10.27, *p* = .002, η_p_^2^ = 0.04; *U* = 8291, *p* = .005, *d* = 0.41.

Finally, we turned to the single item of perceived loyalty. The ANCOVA showed the predicted main effect of agent judgment, *F*(1,480) = 259.78, *p* < .001, η_p_^2^ = 0.35; *U* = 49,147, *p* < .001, *d* = 1.44, and a non-significant but marginal interaction between agent judgment and dilemma type, *F*(1,480) = 3.67, *p* = .056, η_p_^2^ = 0.001. The non-consequentialist was seen as more loyal in both the sacrificial, *F*(1,247) = 149.13, *p* < .001, η_p_^2^ = 0.38; *U* = 13,265, *p* < .001, *d* = 1.52, and impartiality dilemmas, *F*(1,232) = 110.38, *p* < .001, η_p_^2^ = 0.32; *U* = 11,116, *p* < .001, *d* = 1.35, though the effect sizes were slightly larger in the sacrificial dilemma.

#### 4.2.3 Prisoner's Dilemma

In order to assess the effects of agent judgment, dilemma type, and participant judgment in the dilemma on the likelihood that participants made cooperative decisions in the PD we conducted a 2 × 2 × 2 logistic regression model. This model revealed no significant three-way interaction between agent, dilemma, and participant judgment, only a significant interaction of agent judgment and participant judgment, *B* = −1.56, *SE* = 0.62, *Z* = −2.51, *p* = .012, and a marginally significant interaction of agent judgment and dilemma type, *B* = −0.86, *SE* = 0.46, *Z* = −1.88, *p* = .061. These interaction effects were supplemented by a significant main effect of agent judgment, *B* = 1.19, *SE* = 0.33, *Z* = 3.60, *p* < .001, such that across dilemmas and regardless of participant's own judgments, there was more cooperation extended to the non-consequentialist (73%) than the consequentialist (63%) agent. There was, however, no main effect of participant judgment, *B* = 0.08, *SE* = 0.45, *Z* = 0.17, *p* = .86, suggesting that overall, consequentialist participants were roughly as equal to cooperate (77%) as non-consequentialist participants were (64%) - though note that this difference was significant when looking at simple effects with a chi-square analysis ignoring the effects of agent judgment and dilemma type, *x*^2^(1) = 5.79, *p* = .016, with consequentialists more likely to cooperate.

**Fig. 4:**
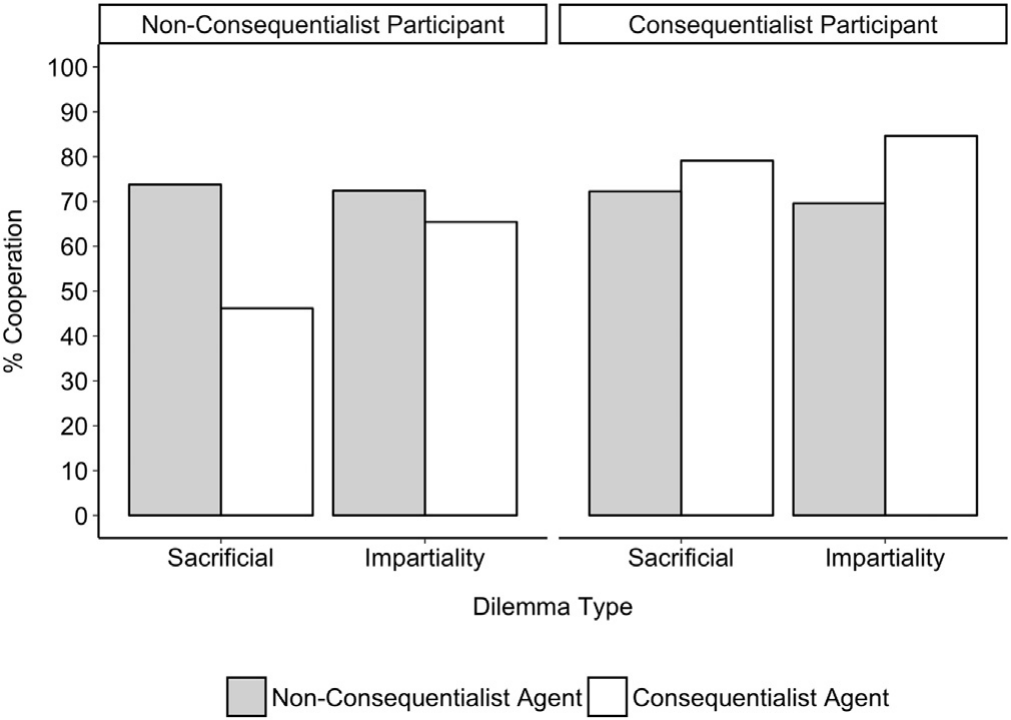
Cooperation in the Prisoner's Dilemma in Study 3 as a function of agent judgment and participant judgment. Across both dilemmas, non-consequentialist participants were more likely to cooperate with the non-consequentialist agent than the consequentialist, but consequentialist participants cooperated equally with both agents.

The significant interaction effect between agent judgment and participant judgment was such that, across dilemmas, non-consequentialist participants were significantly more likely to cooperate with a non-consequentialist agent (73%) than a consequentialist one (55%), *x*^2^(1) = 11.48, *p* < .001, while consequentialist participants were no more likely to cooperate with a consequentialist (81%) or non-consequentialist (72%) agent, *x*^2^(1) = 1.25, *p* = .26 (see Fig. 4). This, we think, explains the simple effect found above of consequentialists being more likely to cooperate than non-consequentialists, when ignoring the effects of dilemma and agent judgment: consequentialist participants do not discriminate, while non-consequentialist participants selectively choose to not cooperate with a consequentialist, thus bringing down overall rates of cooperation.

Finally, while the interaction between agent judgment and dilemma type was only marginally significant, we looked at simple effects regardless and found that across both consequentialist and non-consequentialist participants, people were more likely to cooperate with the non-consequentialist (73%) than the consequentialist agent (57%) in the sacrificial dilemma, *x*^2^(1) = 6.73, *p* = .009. In contrast, in the impartiality dilemma there was equal cooperation extended towards the consequentialist (71%) and non-consequentialist (72%) agents, *x*^2^(1) = 0.02, *p* = .88.

#### 4.2.4 Partner preference

Next, we looked at which agent the participant would have liked to play the PD with, if they'd had a choice. A logistic regression revealed no overall effect of dilemma type, *B* = −0.28, *SE* = 0.19, *Z* = −1.48, *p* = .14 with chi-square analyses revealing that across the dilemmas most participants (65%) preferred to play with a non-consequentialist over a consequentialist (35%). This was the case both in the sacrificial dilemma, with 68% preferring the non-consequentialist, *x*^2^(1) = 33.12, *p* < .001; and the impartiality dilemma, with 62% preferring the non-consequentialist, *x*^2^(1) = 12.93, *p* < .001.

#### 4.2.5 Role suitability

Next, we looked at perceived suitability for different roles (see Fig. 3). For perceived suitability as a friend, an ANCOVA revealed the predicted main effect of agent judgment, *F*(1,480) = 97.14, *p* < .001, η_p_^2^ = 0.17; *U* = 42,377, *p* < .001, *d* = 0.87, along with a significant interaction of dilemma type and agent judgment on how good a friend participants thought the agent would be, *F*(1,480) = 26.17, *p* < .001, η_p_^2^ = 0.05. In both dilemmas when the agent made a non-consequentialist judgment they were expected to make a better friend, but this effect was stronger for the sacrificial dilemma, *F*(1,247) = 117.38, *p* < .001, η_p_^2^ = 0.19; *U* = 12,759, *p* < .001, *d* = 1.34, than for the impartiality dilemma, *F*(1,232) = 10.40, *p* = .001, η_p_^2^ = 0.04; *U* = 8093, *p* = .014, *d* = 0.39.

For perceived suitability as a spouse, an ANCOVA revealed the predicted main effect of agent judgment, *F*(1,480) = 124.95, *p* < .001, η_p_^2^ = 0.21; *U* = 44,134, *p* < .001, *d* = 0.99, along with a significant interaction of dilemma type and agent judgment on how good a spouse participants thought the agent would be, *F*(1,480) = 18.36, *p* < .001, η_p_^2^ = 0.04. In both dilemmas when the agent made a non-consequentialist judgment they were expected to make a better spouse, but this was stronger for the sacrificial dilemma, *F*(1,247) = 119.82, *p* < .001, η_p_^2^ = 0.33; *U* = 12,813, *p* < .001, *d* = 1.35, than for the impartiality dilemma, *F*(1,232) = 22.69, *p* < .001, η_p_^2^ = 0.09; *U* = 9012, *p* < .001, *d* = 0.60.

For suitability as a boss, an ANCOVA revealed the predicted main effect of agent judgment, *F*(1,480) = 47.36, *p* < .001, η_p_^2^ = 0.09; *U* = 38,815, *p* < .001, *d* = 0.63, and a significant interaction of dilemma type and agent judgment on how good a boss participants thought the agent would be, *F*(1,480) = 15.50, *p* < .001, η_p_^2^ = 0.03. When the agent made a non-consequentialist judgment they were expected to make a better boss, but this was stronger again for the sacrificial dilemma *F*(1,247) = 51.14, *p* < .001, η_p_^2^ = 0.17; *U* = 11,316, *p* < .001, *d* = 0.90, than for the impartiality dilemma, *F*(1,232) = 4.68, *p* = .032, η_p_^2^ = 0.02; *U* = 7904, *p* = .036, *d* = 0.28.

Finally for perceived suitability as a political leader, i.e. President of the United States, an ANCOVA revealed the predicted main effect of agent judgment, *F*(1,480) = 8.64, *p* = .003, η_p_^2^ = 0.02; *U* = 33,156, *p* = .013, *d* = 0.29, and a significant interaction of dilemma type and agent judgment on how good a political leader participants thought the agent would be, *F*(1,480) = 8.61, *p* = .004, η_p_^2^ = 0.02. When the agent made a non-consequentialist judgment in the sacrificial dilemma they were expected to make a better political leader, *F*(1,247) = 14.69, *p* < .001, η_p_^2^ = 0.06; *U* = 9693, *p* < .001, *d* = 0.49, but there was no difference in the impartiality dilemma, *F*(1,232) = 0.00, *p* = .99, η_p_^2^ = 0.00; *U* = 6702, *p* = .77, *d* = 0.01.

### 4.3 Discussion

In the context of a sacrificial dilemma, we successfully replicated and extended previous findings. We found that on every single dependent measure, people who made non-consequentialist judgments in a sacrificial dilemma were perceived more positively than those who made a consequentialist judgment. As in Study 2, the pattern of results for the impartiality dilemma was more nuanced, though a definite pattern of preference for the non-consequentialist was observed. Out of 10 dependent measures, the non-consequentialist was preferred in six measures and there was no preference in four measures. The non-consequentialist was thought to be more competent and loyal; thought to make a better friend, spouse, and boss; and preferred as a future partner in a PD. There was no preference, however, in perceptions of morality or warmth, perceived suitability as a political leader, or cooperation extended in a PD.

Our results are equivocal as to whether non-consequentialist or consequentialist participants were actually more cooperative. While neither non-consequentialist nor consequentialist participants reliably cooperated more overall, we did find that while non-consequentialist participants selectively cooperated with non-consequentialist agents, consequentialist participants cooperated equally with both agents. We find no evidence, then, that non-consequentialists are systematically more cooperative than their consequentialist counterparts. That said, it is important to recognize two key caveats. The first caveat is that behaviour in the PD requires both the goal of cooperation and the expectation that others will cooperate (Pruitt & Kimmel, 1977), or in other terms, a social preference to cooperate and a belief that others will too (Everett et al., 2015a, Everett et al., 2015b). We have shown that non-consequentialists distrust a consequentialist agent and do not expect them to cooperate, and this will reduce cooperation. The second caveat is that the cooperation in Study 3 was towards an anonymous stranger with whom the participant will not interact again. Deontological ethics typically focus on the duties and obligations we have, particularly towards those close to us, and it is the respecting of these implicit duties that seems to drive the preference for non-consequentialists (Everett et al., 2016). Given this, even if non-consequentialists are not more cooperative in anonymous settings towards strangers, this does not necessarily indicate that the signal of non-consequentialists being more cooperative is an incorrect one: the dimension in which that we are measuring their cooperation (anonymous one-shot cooperation) is not necessarily the one that the preference for non-consequentialists emerges from (cooperation in repeated contexts with those we have duties or obligations to). It would be interesting, therefore, for future work to explore whether non-consequentialists and consequentialists are more cooperative in different contexts.

## 5 Study 4

In our final study, we wanted to address two potential concerns with the previous studies. The first concern is that there were differences in how much the agents expressed conflict, or recognized competing moral arguments against their decision. The non-consequentialist agent in all dilemmas recognized the conflict by noting they could bring about better consequences, but thought that other moral concerns were more important. Similarly in the impartiality dilemma the consequentialist agent briefly acknowledged the conflicting deontological duties (“by donating the money to charity Amy would have the chance to bring about more happiness for more people, and I think this is more important than any duties she has to her grandson”), but in the sacrificial dilemma the consequentialist agent made no mention of the conflicting duties (“she could find out which is the vaccine and therefore be able to save many more lives, and I think that it is better to save many lives than just one”). It is possible, then, that the strong preference for the non-consequentialist we observed in the sacrificial but not impartial dilemmas was partly driven by the fact that the consequentialist in the sacrificial dilemmas expressed no awareness of moral conflict, but the consequentialist in the impartiality dilemma did (see Table 2 for the manipulation text in Studies 2–3). To address this, in our fourth study we removed the mention of conflict for both agents in both dilemmas (see Table 5 for the text used).

**Table 5:** Reported judgments and justifications of the agent (“Person B”) in Study 4.

	Non-consequentialist agent	Consequentialist agent
Sacrificial (vaccine)	I think that Amy should not inject the lab assistants with the substances to find the vaccine. Killing is just wrong regardless of the consequences.	I think that Amy should inject the lab assistants with the substances to find the vaccine. She could save many more lives - and I think that it is better to save many lives than just one
Impartiality (spending money)	I think that Amy should give the $500 to her grandson so that he could get his car fixed, not the charity. It's most important she respects the duties and obligations she has to her grandson	I think that Amy should give the $500 to the charity providing polio vaccinations in the developing world, not her grandson. It's most important that Amy uses the money to bring about the most happiness for the most people.

The second concern we wanted to address was that in Studies 2–3 we collected data on perceived motives of the agent, but did not report the results in the main manuscript because of issues with the wording of the question (see Footnote 6). We had asked participants to indicate on a 11-point scale how much they thought the agent's decision was driven “more by strategic, reasoned motives versus more empathic, altruistic motives?”. However, on reflection this measure was sub-optimal both because it conflates reasoned and strategic motives (which may not be identical) and forces participants to select one side, when it is possible that participants thought the action is both more strategic or reasoned *and* more altruistic. To address this and gain insight to participants' perceptions of the agent's motives, in Study 4 we used three separate questions to assess perceived motives. First, we had participants rate on a binary scale whether they thought the agent's decision was driven more by emotion or reason (−*5* = *Completely emotion*; +*5* = *completely reason*). Second and third, participants indicate separately how much they thought the agent's decision was driven by strategic and altruistic motives (*1* = *not at all*, *7* = *very much*).

### 5.1 Method

#### 5.1.1 Open science

Our design, hypotheses, and analysis plan were all pre-registered at the Open Science Framework (https://osf.io/xr428/) as part of the Pre-Registration Challenge. We report all measures, manipulations, and exclusions in this study. All data, analysis code, and experiment materials are available for download at: https://osf.io/zf2dp/.

#### 5.1.2 Participants

In accordance with the pre-registration, 500 participants completed the survey online via MTurk. Participants were excluded from completing the survey if they had participated in related studies by us in the past, and were excluded from analysis if they completed the survey more than once (*N* = 2), or failed a simple comprehension check asking them to indicate the decision their partner made in the dilemma (*N* = 41), leaving us with a final sample of 456 participants (218 female; *M*_age_ = 35, *SD* = 10.87). Our sample size was determined through an a priori power analysis (see supplementary methods for details) and a sensitivity power analysis for our main 2 × 2 ANCOVA analysis, assuming an α of 0.05 and power of 0.80, indicated that the minimum effect size we had power to detect was a small effect of *f* = 0.13. All participants were paid $1.20 for participating, in accordance with an hourly US minimum wage of $7.25 and the survey taking approximately 10 min.

#### 5.1.3 Design

This study had a 2 (Dilemma Type: Sacrificial vs. Impartiality) × 2 (Agent Judgment: Non-consequentialist vs. Consequentialist) between-subjects experimental design, and the procedure was the same as in Study 2, except without an economic game and the addition of the three motives questions outlined in the introduction to this study.

#### 5.1.4 Analysis plan

The analysis plan for this study was identical to that in Studies 2–3. See Table 6 for *M*s and *SD*s as a function of dilemma type and agent judgment, as well as *p*-values from a Mann-Whitney *U* test, and effect sizes. Again, see supplementary results for analyses looking at a 2 × 2 × 2 ANOVA in which we entered participant moral judgment as a fixed factor instead of a covariate, though like in the previous studies there were no significant 3-way interactions.

**Table 6:** *M*s, *SD*s, *p*-values and effects sizes in Study 4 as a function of dilemma type and agent judgment.

	Sacrificial dilemmas					Impartiality dilemmas				
	Non-conseq.	Consq.	*p* Value	*d*	Prefer	Non-conseq.	Consq.	*p* Value	*d*	Prefer
Morality	5.48 (1.27)	4.48 (1.45)	<.001	0.74	Non-C	5.40 (1.12)	5.06 (1.22)	.050	0.29	Non-C
Warmth	5.45 (1.10)	4.55 (1.36)	<.001	0.74	Non-C	5.06 (1.06)	4.93 (1.19)	.47	0.11	–
Competence	4.99 (1.41)	5.00 (1.30)	.99	0.01	–	5.32 (1.14)	5.02 (1.13)	.032	0.27	Consq
Loyalty	5.51 (1.32)	4.40 (1.61)	<.001	0.77	Non-C	6.08 (1.19)	4.32 (1.62)	<.001	1.24	Non-C
Suitability as a friend	5.46 (1.26)	4.38 (1.56)	<.001	0.77	Non-C	5.38 (1.34)	4.54 (1.54)	<.001	0.58	Non-C
Suitability as a spouse	5.15 (1.42)	4.25 (1.61)	<.001	0.60	Non-C	5.28 (1.37)	4.31 (1.56)	<.001	0.67	Non-C
Suitability as a boss	4.80 (1.56)	4.48 (1.79)	.29	0.19	–	4.86 (1.32)	4.68 (1.51)	.39	0.13	–
Suitability as a political leader	4.07 (1.87)	4.30 (1.83)	.29	0.13	–	4.16 (1.61)	4.43 (1.67)	.12	0.16	–
Emotion (−) vs reason (+)	−1.45 (3.14)	2.63 (2.48)	<.001	1.42	a	0.20 (2.93)	0.68 (3.30)	.15	0.15	–
Strategic motives	2.43 (1.43)	5.38 (1.46)	<.001	2.04	Consq	3.85 (1.78)	4.14 (1.81)	.23	0.16	–
Altruistic motives	4.68 (1.70)	4.80 (1.51)	.93	0.07	–	4.08 (1.53)	5.18 (1.36)	<.001	0.76	Consq

a The consequentialist was expected to be more driven by reason, and the non-consequentialist was thought to be more driven by emotion.

### 5.2 Results

#### 5.2.1 Participant judgments

The majority of participants endorsed the non-consequentialist option in the sacrificial dilemmas (59%), rejecting the sacrifice of one to save the lives of a greater number. Similarly, most participants endorsed the non-consequentialist option in the impartiality dilemmas (85%), endorsing helping a family member over impartially helping a greater number. Again, further results looking at participant judgment can be found in the supplementary materials.

#### 5.2.2 Character ratings

We first looked at character ratings (see Fig. 1). For the two items measuring perceived morality (α = 0.89), the ANCOVA revealed the predicted main effect of agent judgment, *F*(1,451) = 34.29, *p* < .001, η_p_^2^ = 0.07; *U* = 33,548, *p* < .001, *d* = 0.54, and a significant interaction of dilemma type and agent judgment on how moral participants perceived the agent to be, *F*(1,451) = 8.54, *p* = .004, η_p_^2^ = 0.02. The non-consequentialist was seen as more moral than the consequentialist in both the sacrificial dilemma, *F*(1,226) = 32.58, *p* < .001, η_p_^2^ = 0.13; *U* = 9165, *p* < .001, *d* = 0.74, and the impartiality dilemma, *F*(1,224) = 5.27, *p* = .023, η_p_^2^ = 0.02; *U* = 7397, *p* = .050, *d* = 0.29, though the effect was stronger in the sacrificial dilemma.

For the two items measuring perceived warmth (α = 0.77), the ANCOVA revealed the predicted main effect of agent judgment, *F*(1,451) = 23.33, *p* < .001, η_p_^2^ = 0.05; *U* = 31,901, *p* < .001, *d* = 0.42, and a significant interaction of dilemma type and agent judgment on how warm and sociable participants perceived the agent to be, *F*(1,451) = 12.59, *p* < .001, η_p_^2^ = 0.03. While a non-consequentialist was seen as warmer than the consequentialist in the sacrificial dilemma, *F*(1,226) = 32.19, *p* < .001, η_p_^2^ = 0.12; *U* = 8996, *p* < .001, *d* = 0.74, there was no difference in the impartiality dilemma, *F*(1,234) = 0.95, *p* = .33, η_p_^2^ = 0.00; *U* = 6795, *p* = .47, *d* = 0.11.

For the two items measuring perceived competence (α = 0.89), the ANCOVA showed no main effect of agent judgment, *F*(1,451) = 1.99, *p* = .16, η_p_^2^ = 0.00; *U* = 28,134, *p* = .12, *d* = 0.13, and no interaction between agent judgment and dilemma type, *F*(1,451) = 1.52, *p* = .22, η_p_^2^ = 0.00. Contrary to predictions, there was no difference in the sacrificial dilemma, *F*(1,226) = 0.00, *p* = .95, η_p_^2^ = 0.00; *U* = 6473, *p* = .99, *d* = 0.01, but the non-consequentialist was seen as more competent in the impartiality dilemma, *F*(1,224) = 4.48, *p* = .035, η_p_^2^ = 0.02; *U* = 7488, *p* = .032, *d* = 0.27.

Finally, we turned to the single item of perceived loyalty. The ANCOVA showed the predicted main effect of agent judgment, *F*(1,451) = 122.74, *p* < .001, η_p_^2^ = 0.21; *U* = 39,389, *p* < .001, *d* = 1.01, and a significant interaction between agent judgment and dilemma type, *F*(1,451) = 4.78, *p* = .029, η_p_^2^ = 0.01. The non-consequentialist was seen as more loyal in both the sacrificial, *F*(1,226) = 37.28, *p* < .001, η_p_^2^ = 0.14; *U* = 9081, *p* < .001, *d* = 0.77, and impartiality dilemmas, *F*(1,224) = 92.72, *p* < .001, η_p_^2^ = 0.29; *U* = 10,403, *p* < .001, *d* = 1.24, though the effect was stronger in the impartiality dilemma (see Fig. 2).

#### 5.2.3 Role suitability

Next, we looked at perceived suitability for different roles (see Fig. 3). For perceived suitability as a friend, an ANCOVA revealed the predicted main effect of agent judgment, *F*(1,451) = 55.12, *p* < .001, η_p_^2^ = 0.11; *U* = 35,363, *p* < .001, *d* = 0.68, and no interaction effect, *F*(1,451) = 1.13, *p* = .29, η_p_^2^ = 0.00. In both dilemmas when the agent made a non-consequentialist judgment they were expected to make a better friend (sacrificial dilemma: *F*(1,226) = 34.59, *p* < .001, η_p_^2^ = 0.13; *U* = 9094, *p* < .001, *d* = 0.77; impartiality dilemma: *F*(1,224) = 21.10, *p* < .001, η_p_^2^ = 0.09; *U* = 8494, *p* < .001, *d* = 0.58).

For perceived suitability as a spouse, an ANCOVA revealed the predicted main effect of agent judgment, *F*(1,451) = 48.53, *p* < .001, η_p_^2^ = 0.10; *U* = 34,817, *p* < .001, *d* = 0.64, but no significant interaction, *F*(1,451) = 0.02, *p* = .90, η_p_^2^ = 0.00. The non-consequentialist agent was thought to make a better spouse in both the sacrificial, *F*(1,226) = 21.81, *p* < .001, η_p_^2^ = 0.09; *U* = 8589, *p* < .001, *d* = 0.60, and the impartiality dilemma, *F*(1,224) = 26.83, *p* < .001, η_p_^2^ = 0.11; *U* = 8706, *p* < .001, *d* = 0.67.

For suitability as a boss, an ANCOVA revealed no main effect of agent judgment, *F*(1,451) = 3.58, *p* = .059, η_p_^2^ = 0.01; *U* = 27,809, *p* = .17, *d* = 0.17, and no interaction of dilemma type and agent judgment, *F*(1,451) = 0.40, *p* = .53, η_p_^2^ = 0.00. Similarly for perceived suitability as a political leader, an ANCOVA revealed no main effect of agent judgment, *F*(1,451) = 1.81, *p* = .018, η_p_^2^ = 0.00; *U* = 23,441, *p* = .071, *d* = 0.14, and no interaction of dilemma type and agent judgment, *F*(1,451) = 0.04, *p* = .84, η_p_^2^ = 0.00.

#### 5.2.4 Perceived motives

First looking at perceptions of whether the agents' decision was driven more by emotion or reason on binary scale, we observed a significant main effect of agent judgment, *F*(1,451) = 65.63, *p* < .001, η_p_^2^ = 0.13; *U* = 15,222, *p* < .001, *d* = 0.74, and a significant interaction of dilemma type and agent judgment, *F*(1,451) = 41.39, *p* < .001, η_p_^2^ = 0.08. In the sacrificial dilemma the non-consequentialist was thought as being more driven by emotion and the consequentialist by reason, *F*(1,226) = 118.54, *p* < .001, η_p_^2^ = 0.34; *U* = 2185, *p* < .001, *d* = 1.42, but there was no difference in the impartiality dilemma, *F*(1,224) = 1.19, *p* = .28, η_p_^2^ = 0.01; *U* = 5724, *p* = .15, *d* = 0.15.

**Fig. 5:**
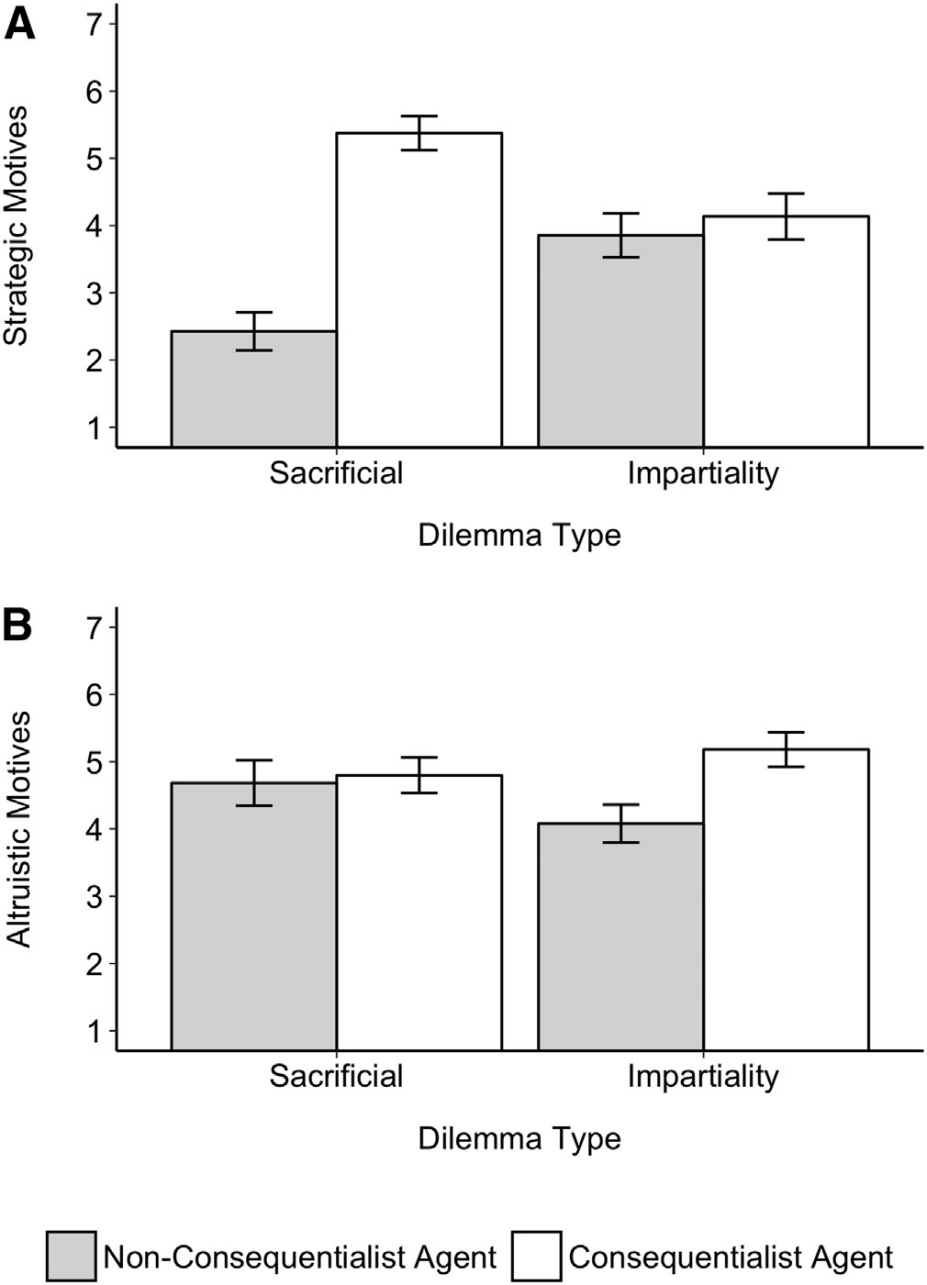
Perceived motives in Study 4 as a function of agent judgment and participant judgment. Results show that in the sacrificial dilemma, the consequentialist was seen as being driven more by strategic motives (5a) than the non-consequentialist, and in the impartiality dilemma the consequentialist was seen as driven more by altruistic motives (5b) than the non-consequentialist. Error bars represent 95% confidence intervals.

Next, we looked at how much the agent's decision was thought to be influenced by strategic motives. We observed a significant main effect of agent judgment, *F*(1,451) = 107.04, *p* < .001, η_p_^2^ = 0.19; *U* = 13,549, *p* < .001, *d* = 0.92, and a significant interaction of dilemma type and agent judgment, *F*(1,451) = 74.39, *p* < .001, η_p_^2^ = 0.14. In the sacrificial dilemma the consequentialist was thought as being more driven by strategic motives than the non-consequentialist, *F*(1,226) = 227.48, *p* < .001, η_p_^2^ = 0.50; *U* = 1235, *p* < .001, *d* = 2.04, but there was no difference in the impartiality dilemma, *F*(1,224) = 1.14, *p* = .29, η_p_^2^ = 0.01; *U* = 5859, *p* = .23, *d* = 0.16 (See Fig. 5).

Finally, we looked at how much the agent's decision was thought to be influenced by altruistic motives. We observed a significant main effect of agent judgment, *F*(1,451) = 17.96, *p* < .001, η_p_^2^ = 0.04; *U* = 20,562, *p* < .001, *d* = 0.40, and a significant interaction of dilemma type and agent judgment, *F*(1,451) = 11.73, *p* < .001, η_p_^2^ = 0.03. In the sacrificial dilemma there was no difference in how much either agent was thought to be influenced by altruistic motives, *F*(1,226) = 0.33, *p* = .56, η_p_^2^ = 0.00; *U* = 6421, *p* = .93, *d* = 0.07, but in the impartiality dilemma the consequentialist was thought to be more driven by altruistic motives, *F*(1,224) = 32.43, *p* < .001, η_p_^2^ = 0.13; *U* = 3866, *p* < .001, *d* = 0.76.

### 5.3 Discussion

In Study 4 we conducted another pre-registered investigation of perceptions of non-consequentialist and consequentialist agents in both sacrificial and impartiality dilemmas, adding three new questions to assess perceptions of the agent's motives and addressing potential concerns with the manipulation of the agent's judgment.

First, we saw that the consequentialist was seen as more influenced by strategic motives in the sacrificial dilemma, but more by altruistic motives in the impartiality dilemma. And while in the sacrificial dilemma the non-consequentialist was thought as being more driven by emotion and the consequentialist by reason, there was no difference in the impartiality dilemmas for perceptions of being driven by reason or emotions.

Second, we addressed the potential concern with the previous studies that the consequentialist expressed awareness of conflict for the impartiality, but not sacrificial, dilemma. When ensuring that all agents expressed no recognition of competing moral reasons, we broadly replicated the findings of Study 3, with some minor differences. Like in Study 3, in the impartiality dilemma the non-consequentialist was seen as more competent, more loyal, and expected to make a better friend and spouse. However, while in Study 3 the non-consequentialist was thought to make a better boss, there was no difference in Study 4; and while in Study 3 there was no difference in perceived morality, in Study 4 the non-consequentialist was seen as more moral. In neither study was there a difference in perceptions of warmth or suitability as a political leader. It seems clear, then, that our pattern of results cannot be explained simply by the agent's (lack of) expression of moral conflict: regardless of whether they expressed conflict or not, the non-consequentialist in the impartiality dilemma tended to be favored for direct, interpersonal roles. This mirrors our previous work showing that expressing internal conflict through reported emotional difficulty when making a consequentialist decision reduces, but does not fully eliminate, the preference for a non-consequentialist over a consequentialist (Everett et al., 2016). It will be interesting for future research to examine how expressing conflict through different kinds of moral justifications influences person perception.

## 6 General discussion

Much work over the last decade has focused on the psychological processes underlying judgments about whether it is moral to sacrifice one innocent person to save a greater number of people. In recent years, befitting the fundamentally social role of moral judgments, researchers have begun to consider the social consequences of these judgments: how are consequentialist individuals who endorse harming for the greater good perceived? Previous work has shown that the judgment a person makes in these sacrificial dilemmas influences how moral, warm, and competent they are perceived to be, and even how much cooperation is extended towards them in economic games (e.g. Bostyn & Roets, 2017; Everett et al., 2016; Rom et al., 2017; Uhlmann et al., 2013). But while this work has focused solely on judgments in sacrificial dilemmas and has been thought to shed light on the social consequences of consequentialist judgments in general, consequentialism involves much more than just judgments about whether to sacrifice one to save a greater number.

As outlined in the two-dimensional model of utilitarian psychology (Kahane et al., 2018, Kahane et al., 2015), judgments in sacrificial dilemmas tap the endorsement of “*instrumental harm*”, which can be theoretically and empirically distinguished from impartiality dilemmas that tap the more positive, impartial welfare-maximising dimension (“*impartial beneficence*”) of consequentialist theories and consequentialist tendencies in ordinary people. Because this previous work on perception of consequentialist agents has focused almost exclusively on sacrificial dilemmas, it has remained unknown whether the preference for non-consequentialists over consequentialists operates similarly in impartiality dilemmas in which someone faces the decision to help someone close to them or a greater number of strangers.

In four studies, we investigated perceptions of consequentialist and non-consequentialist decision makers in both sacrificial dilemmas tapping instrumental harm, and impartiality dilemmas tapping impartial beneficence. Pre-registering our analyses and predictions, we included the most comprehensive range of dependent measures used in this literature to date, using different economic games (the Trust Game and the Prisoner's Dilemma); examining the distinct dimensions along which the agent's character could be perceived (warmth; competence; morality); exploring the different processes or motivations perceived to influence the agent's moral decision (strategic, altruistic, emotion-based, and reason-based); and considering the different social roles and relationships in which the agent would be preferred (as a friend, a spouse, a boss, and as a political leader).

### 6.1 Person perception in sacrificial dilemmas

In the domain of sacrificial harm, our findings strongly confirm previous work highlighting the cost of being consequentialist. We show in three studies that non-consequentialists were consistently preferred over consequentialists. We argue that this is perfectly explicable on a partner choice account of non-consequentialist moral intuitions (Everett et al., 2016). The consequentialist rejection of any constraints on the maximisation of welfare means that if killing one's partner maximises the greater good, then that is what one should do. And yet when selecting a social partner for the purposes of continued cooperative exchange, such a person would seem disastrous. Instead, we have argued, it would be more advantageous for people to seek social partners who maximise good consequences, but also those who also exhibit respect for rights, duties, and the individuality of persons. Given that consequentialists are consistently disfavored in the cooperation market, partner choice mechanisms could explain why our moral intuitions in such sacrificial dilemmas so often lean deontological (Everett et al., 2016). Put simply, non-consequentialist, or deontological, judgments confer an adaptive function by increasing the likelihood of being chosen as a cooperation partner. It pays – in our studies, literally – to be a non-consequentialist in a sacrificial dilemma.

### 6.2 Person perception in impartiality dilemmas

What about impartiality dilemmas? As discussed in the introduction, how people are perceived in the domain of impartial beneficence remains largely unknown, because almost all previous work has focused on sacrificial dilemmas. The pattern of results in the impartiality dilemmas was much more nuanced than the unequivocal preference for the non-consequentialist in the sacrificial dilemmas, though simply counting overall results suggest that the non-consequentialist has the edge over the consequentialist in the domain of impartial beneficence too: overall, we saw few cases where the consequentialist was preferred, typically seeing either a null effect or that the non-consequentialist was favored. Most interestingly, our results using the impartiality dilemmas appear to suggest a predictable pattern of when non-consequentialists are preferred – and when they are not.

Impartial consequentialists were consistently disfavored for roles involving a direct interpersonal relationship, even if they were not explicitly rated as being deficient in morality and warmth. In all four studies, the impartial consequentialist was thought to be less loyal and thought to make a worse friend and spouse, even if they were not always explicitly rated as being deficient in morality and warmth. And in Study 3 - but not Study 2 - the impartial consequentialist was also disfavored as a future partner in a Prisoner's Dilemma and was thought to make a worse boss. This makes sense given the theoretical basis we draw on in the introduction: consequentialism's requirement for the impartial maximisation of welfare is often inconsistent with the nature of special relationships like friendship and familial duties that are a fundamental part of common-sense morality (Jeske, 2014; W.D. Ross, 1930). When we enter a close, interpersonal partnership – with a friend, or a team-mate, or a spouse - we expect partiality by agreeing on certain special obligations ourselves and expecting them to be honoured by our friend. We are expected to help our friend when they need it, and we expect our friend to help us when we need it, but consequentialism's denial of any such obligations is incompatible with what we seek in social partners. This, we think, is why participants consistently rated the impartial consequentialist as being less loyal and as making a worse friend or spouse.

While it makes sense for non-consequentialists to be favored for direct, interpersonal relationships, it is much more reasonable – even preferable - to favor a consequentialist for distant, impersonal roles like a political leader, and this what we what found in Studies 1 and 2. The job of an effective political leader can be plausibly described as to make constituents better off, and part of this requires acting impartially to not favor one's own self-interest or the interest of one's immediate family. Indeed, recent work has shown that people do not endorse efficient maximisation in charitable giving unless one is in a position of responsibility, like a political leader (Berman, Barasch, Levine, & Small, 2018). Of course, this is not to say we should prefer an absolutely impartial political leader: a national leader who neglects his own country's well-being and finances to support a poorer developing country is hardly likely to be lauded as a moral paragon. We might prefer, in other words, political leaders who are impartial within our group, but partial between groups (and indeed, other research from social psychology demonstrates that intergroup partiality is expected and favored in group leaders (e.g. Duck and Fielding, 1999, Duck and Fielding, 2003)). It would be fruitful for future work to examine this directly, exploring in more detail the way that moral impartiality is favored in both intra- and inter-group contexts with varying degrees of personal connection.

Overall, then, while the pattern of results is weaker than for sacrificial dilemmas, a tentative conclusion can be drawn: there may be some costs of being consequentialist in the domain of impartial beneficence, and this is especially manifested when considering suitability and desirability for direct, personal relationships.

### 6.3 Person perception from impartial beneficence vs. instrumental harm

It is unsurprising that we saw stronger preferences for a non-consequentialist agent who refuses to *harm* for the greater good, compared to a non-consequentialist agent who refuses to *help* for the greater good. There are of course important differences – both theoretical and psychological - between instrumentally killing someone to achieve the greater good and impartially maximising welfare by privileging strangers over our family (Kahane et al., 2018). Many deontological traditions distinguish between the “perfect” absolute and universal duties we have to refrain from some acts – e.g. murder -, and the more “imperfect” and context-dependent duties we have to help others (e.g. Kant, 1797/2002). Analogously to research on punishment and minimal and maximal standards in social psychology (Kessler et al., 2010), in non-consequentialist moral approaches we can always blame someone for murder, but we cannot always blame them for not helping others. This is demonstrated psychologically with the “omission bias”, by which directly harming someone is usually perceived as morally worse than failing to help them (Baron & Ritov, 1994; Ritov & Baron, 1990; Spranca et al., 1991; see also Siegel et al., 2017). When required to maximise the greater good, a consequentialist endorsing instrumental harm may harm or even kill their partner to bring about an impartially better state of affairs, while a consequentialist endorsing impartial beneficence may simply neglect to help their partner, and it is reasonable to assume that consequentialists who directly harm will be perceived more negatively than those who just fail to help.

Furthermore, much psychological research has documented a positive-negative asymmetry in social cognition in general (Baumeister et al., 2001) and person perception in particular (Skowronski & Carlston, 1989). In general, “bad is stronger than good” such that bad emotions are more powerful than good emotions, bad information is processed more thoroughly than good information, and – most critically - bad information about a person is more impactful than good information about a person. It is reasonable, then, that information about a ‘bad’ action (murder) would be more impactful than information about different types of ‘good’ actions (helping others). It will be interesting for future research to explore more the way that people performing obligatory and supererogatory impartially beneficent acts will be seen.

### 6.4 The costs of consequentialism: Implications for the partner choice model

We have previously argued for a partner choice^7^ model of non-consequentialist moral intuitions in sacrificial dilemmas (Everett et al., 2016), whereby if non-consequentialist agents are preferred as social partners and are therefore more likely to reap the benefits of cooperation, consequentialist moral intuitions could therefore have become disfavored over more deontological, non-consequentialist ones. Replicating previous work and supporting such a partner choice model, we found again here that non-consequentialists in sacrificial dilemmas were indeed favored, being both cooperated more with a TG and a PD, and being more likely to be selected as a partner PD or TG.

In the impartiality dilemmas, however, we found little direct support for a partner choice model in the context of anonymous economic exchanges: Participants did not cooperate more with non-consequentialists. While these null-results are problematic for the partner choice model of moral intuitions, we think that it's not possible to completely reject this account, even as it applies to the domain of impartial beneficence. Critically, it is important to distinguish between a partner choice model of moral intuitions in the domain of instrumental harm from one for impartial beneficence. These two dimensions are both theoretically and empirically dissociable, each having distinct psychological correlates and appearing to be driven by distinct psychological processes (Kahane et al., 2018, Kahane et al., 2015). Proto-consequentialist tendencies are not a unitary phenomenon and so it is perfectly plausible that partner choice mechanisms would have favored non-consequentialist intuitions in the domain of instrumental harm but not impartial beneficence. But even recognizing this, we think that there are two key reasons why even within the domain of impartial beneficence, there are still reasons to think the partner choice model might hold.

First, we acknowledge that the anonymous economic game context may not be ideal to study partner preferences in the context of impartial beneficence: the consequentialist agents explicitly indicated they thought it better to impartially maximise welfare even with strangers, and participants were themselves strangers to the agent. And while they seemed to understand that they stood to benefit from the agent's impartial beneficence in an anonymous economic exchange, they *still* did not cooperate more with them. It is possible, then, that a tension between participants' self-interest (which should lead towards to cooperating more with a consequentialist) and their preference for non-consequentialists in interpersonal interactions (c.f. the other results) cancelled each other out. When playing a game in which the implicit social contract is stronger (for example through players knowing each other), it is possible that we would see a preference for the non-consequentialist.

Second, even if participants did not cooperate more with either agent in the anonymous economic games, they did consistently rate the non-consequentialist as being a better friend and spouse, in line with previous evidence that non-consequentialists in sacrificial dilemmas are favored as long-term mating partners (Brown & Sacco, 2017). Given that most people do tend to be more prosocial towards their close friends and family, if in real life non-consequentialists were preferred as friends and spouses, this would also lead to the same partner choice mechanisms occurring.

### 6.5 Practical implications

Finally, our work has practical implications concerning how in everyday life groups and individuals who advocate a more impartial, welfare-maximising consequentialist approach to moral decisions – such as the “effective altruism” movement – might expect to be perceived, and how this might limit their advocacy. Peter Singer is the most influential living utilitarian thinker, and in part because of his utilitarian consequentialist moral views is a leading proponent of effective altruism: a movement built around the idea of using reason and evidence to find the best ways to help others. In particular, effective altruism is concerned with using one's resources to have the most impact in helping others, and many effective altruists have pledged to give at least 10% of their income to cost-effective charities (MacAskill, 2015; Singer, 2015). In practice, this means that effective altruists advocate for donating money to charity to help relieve poverty and disease in the developing world (e.g. the Against Malaria Foundation) rather than advocating for local but less effective charities (e.g. local community centers for the elderly or disabled). In this way, the consequentialist action in our impartiality dilemmas we used in this study – and especially the spending money variant – directly match onto the action that we should do on effective altruism principles. Our work suggests that effective altruists may face stumbling blocks in how they are perceived by others and that, while receiving far less criticism than consequentialists can receive for endorsing instrumental harm, this may still have harmful consequences for how the movement is perceived and therefore, presumably, how many people join the movement and adopt its principles.

## Footnotes

1 We report all measures in Study 1, with one exception: as an exploratory question for different purposes, we asked participants how much they intended to vote for Donald Trump or Hilary Clinton in the then-ongoing US elections. These were not relevant to our purposes here, and are not analyzed or reported here (or elsewhere).

2 There were a couple of differences between the results from each dilemma in Study 1, but the pattern was still the same. For example, in both the spending time and spending money variants, the consequentialist was thought to make a better political leader, but this was only statistically significant in the spending money dilemma.

3 We originally also had a third dilemma involving “sharing opportunities”, but for multiple theoretical and empirical issues outlined in the supplementary file for Study 1, we excluded this third dilemma from analysis and did not include it in the subsequent pre-registered studies.

4 We also ran more exploratory analyses using scores on the Oxford Utilitarianism Scale as a covariate instead of participant wrongness of the specific consequentialist action in the dilemma. Across the dependent measures, these two analyses yielded largely identical results and so we report only the primary (pre-registered, for Studies 2–3) analysis using wrongness ratings in the dilemma.

5 We also collected data asking participants to indicate on a single scale how much they thought the agent's “decision was driven more by strategic, reasoned motives [−5] versus more empathic, altruistic motives [+5]?”. However, on reflection this measure is sub-optimal both because it conflates reasoned and strategic motives, perhaps inappropriately, and forces participants to select one side (perhaps participants think the action is both more reasoned and altruistic). Given these methodological concerns, we do not report these results in the paper.

6 For both dilemmas we changed the protagonist's name to Amy so that it was the same for all participants. For “spending money” dilemma, we reduced the amount from $2000 to $500, because we were concerned that $2000 was unrealistic as a cost for fixing a car, since this would likely buy a new car. For the “spending time” dilemma we changed the charity from the Against Malaria Foundation to one that provides polio vaccinations to children in developing countries, because we thought that more people would know about polio than the work of the AMF, and we changed the reason that the mother needed help from feeling lonely to instead because she was suffering from migraines, to make both causes related to health.

7 It should be noted that our effects can also be seen as consistent with partner *control* models, though partner choice and partner control mechanisms often work in tandem and for the purposes of our arguments here lead to convergent predictions. The key idea that expressing consequentialist moral judgments incurs a social cost remains the same: the difference is whether at a given time this social cost is manifested through an individual attempting to change their partner's behaviour (partner control) or just change to another partner (partner choice).

## References

[bb0005] Annis D.B. (1987). The meaning, value, and duties of friendship. American Philosophical Quarterly.

[bb0010] Axelrod R. (1980). More effective choice in the prisoner's dilemma. Journal of Conflict Resolution.

[bb0015] Baron J., Ritov I. (1994). Reference points and omission bias. Organizational Behavior and Human Decision Processes.

[bb2000] Baumard N., André J.B., Sperber D. (2013). A mutualistic approach to morality: The evolution of fairness by partner choice. Behavioral and Brain Sciences.

[bb0020] Baumeister R.F., Bratslavsky E., Finkenauer C., Vohs K.D. (2001). Bad is stronger than good. Review of General Psychology.

[bb0025] Bentham J. (1983). The collected works of Jeremy Bentham: Deontology, together with a table of the springs of action; and the article on utilitarianism.

[bb0030] Berg J., Dickhaut J., McCabe K. (1995). Trust, reciprocity, and social history. Games and Economic Behavior.

[bb4500] Berman J.Z., Barasch A., Levine E.E., Small D.A. (2018). Impediments to Effective Altruism: The Role of Subjective Preferences in Charitable Giving. Psychological Science.

[bb0040] Bostyn D.H., Roets A. (2017). Trust, trolleys and social dilemmas: A replication study. Journal of Experimental Psychology. General.

[bb7000] Choshen-Hillel S., Shaw A., Caruso E.M. (2015). Waste management: How reducing partiality can promote efficient resource allocation. Journal of Personality and Social Psychology.

[bb0055] Cocking D., Oakley J. (1995). Indirect consequentialism, friendship, and the problem of alienation. Ethics.

[bb1500] Dawes R.M. (1980). Social dilemmas. Annual Review of Psychology.

[bb5500] Duck J.M., Fielding K.S. (1999). Leaders and subgroups: One of us or one of them?. Group Processes & Intergroup Relations.

[bb6500] Duck J.M., Fielding K.S. (2003). Leaders and their treatment of subgroups: Implications for evaluations of the leader and the superordinate group. European Journal of Social Psychology.

[bb8000] Dungan J., Waytz A., Young L. (2014). Corruption in the context of moral trade-offs. Journal of Interdisciplinary Economics.

[bb0090] Everett J.A.C., Faber N.S., Crockett M. (2015). Preferences and beliefs in ingroup favoritism. Frontiers in Behavioral Neuroscience.

[bb0095] Everett J.A.C., Faber N.S., Crockett M.J. (2015). The influence of social preferences and reputational concerns on intergroup prosocial behaviour in gains and losses contexts. Royal Society Open Science.

[bb0100] Everett J.A.C., Ingbretsen Z., Cushman F., Cikara M. (2017). Deliberation erodes cooperative behavior — Even towards competitive out-groups, even when using a control condition, and even when eliminating selection bias. Journal of Experimental Social Psychology.

[bb0105] Everett J.A.C., Pizarro D.A., Crockett M.J. (2016). Inference of trustworthiness from intuitive moral judgments. Journal of Experimental Psychology. General.

[bb4000] Fehr E., Schmidt K.M. (1999). A theory of fairness, competition, and cooperation. The Quarterly Journal of Economics.

[bb0130] Fried C. (1978). Right and wrong.

[bb2500] Hardin G. (1968). The tragedy of the commons. Science.

[bb3500] Hardin G. (1998). Extensions of "the tragedy of the commons". Science.

[bb0145] Held V. (2006). The ethics of care: Personal, political, and global.

[bb0155] Hughes J.S. (2017). In a moral dilemma, choose the one you love: Impartial actors are seen as less moral than partial ones. British Journal of Social Psychology.

[bb0160] Jeske D., Zalta E.N. (2014). Special obligations. The Stanford encyclopedia of philosophy (Spring 2014).

[bb0170] Kahane G., Everett J.A.C., Earp B.D., Caviola L., Faber N.S., Crockett M.J., Savulescu J. (2018). Beyond sacrificial harm: A two-dimensional model of utilitarian psychology. Psychological Review.

[bb0175] Kahane G., Everett J.A.C., Earp B.D., Farias M., Savulescu J. (2015). “Utilitarian” judgments in sacrificial moral dilemmas do not reflect impartial concern for the greater good. Cognition.

[bb0185] Kamm F.M. (2007). Intricate ethics: Rights, responsibilities, and permissible harm.

[bb0190] Kant I. (2002). Groundwork for the metaphysics of morals.

[bb7500] Kessler T., Neumann J., Mummendey A., Berthold A., Schubert T., Waldzus S. (2010). How do we assign punishment? The impact of minimal and maximal standards on the evaluation of deviants. Personality and Social Psychology Bulletin.

[bb8500] MacAskill W. (2015). Doing good better: Effective altruism and a radical new way to make a difference.

[bb0210] Mill J.S. (1863). Utilitarianism.

[bb3000] Noë R., Hammerstein P. (1994). Biological markets: supply and demand determine the effect of partner choice in cooperation, mutualism and mating. Behavioral Ecology and Sociobiology.

[bb0215] Pruitt D.G., Kimmel M.J. (1977). Twenty years of experimental gaming: Critique, synthesis, and suggestions for the future. Annual Review of Psychology.

[bb0225] Rapoport A., Chammah A.M. (1965). Prisoner's dilemma: A study in conflict and cooperation.

[bb0230] Rawls J. (1971). A theory of justice.

[bb0235] Ritov I., Baron J. (1990). Reluctance to vaccinate: Omission bias and ambiguity. Journal of Behavioral Decision Making.

[bb0240] Rom S.C., Weiss A., Conway P. (2017). Judging those who judge: Perceivers infer the roles of affect and cognition underpinning others' moral dilemma responses. Journal of Experimental Social Psychology.

[bb0245] Ross W.D. (1930). The right and the good.

[bb1000] Sacco D.F., Brown M., Lustgraaf C.J., Hugenberg K. (2017). The adaptive utility of deontology: Deontological moral decision-making fosters perceptions of trust and likeability. Evolutionary Psychological Science.

[bb0250] Scanlon T.M. (1998). What we owe to each other.

[bb5000] Shaw A. (2013). Beyond “to share or not to share” The impartiality account of fairness. Current Directions in Psychological Science.

[bb0260] Siegel J.Z., Crockett M.J., Dolan R.J. (2017). Inferences about moral character moderate the impact of consequences on blame and praise. Cognition.

[bb9500] Singer P. (2015). The most good you can do: How effective altruism is changing ideas about living ethically.

[bb0265] Spranca M., Minsk E., Baron J. (1991). Omission and commission in judgment and choice. Journal of Experimental Social Psychology.

[bb6000] Tyler T.R. (2000). Social justice: Outcome and procedure. International Journal of Psychiatry.

[bb0280] Uhlmann E.L., Zhu L.(.L.)., Tannenbaum D. (2013). When it takes a bad person to do the right thing. Cognition.

[bb9000] Waytz A., Dungan J., Young L. (2013). The whistleblower's dilemma and the fairness–loyalty tradeoff. Journal of Experimental Social Psychology.

[bb0295] Woodcock S. (2009). When will your consequentialist friend abandon you for the greater good. Journal of Ethics and Social Philosophy.

